# Durable Proton Exchange Membrane Based on Polymers of Intrinsic Microporosity for Fuel Cells

**DOI:** 10.1002/adma.202419534

**Published:** 2025-03-27

**Authors:** Xiaochen Yang, Zhiming Feng, Mustafa Alshurafa, Ming Yu, Andrew B. Foster, Heng Zhai, Tianmu Yuan, Yiheng Xiao, Carmine D'Agostino, Ling Ai, Maria Perez‐Page, Keenan Smith, Fabrizia Foglia, Adam Lovett, Thomas S. Miller, Jianuo Chen, Peter M. Budd, Stuart M. Holmes

**Affiliations:** ^1^ Department of Chemical Engineering The University of Manchester Manchester M13 9PL UK; ^2^ Department of Chemistry The University of Manchester Manchester M13 9PL UK; ^3^ Department of Chemical Engineering The University of Melbourne Melbourne VIC 3010 Australia; ^4^ Department of Chemistry University College London London WC1H 0AJ UK; ^5^ Department of Chemical Engineering University College London London WC1E 7JE UK

**Keywords:** fuel cell, microporous structure, phosphoric acid, PIM‐1, proton exchange membrane

## Abstract

High‐temperature proton exchange membrane fuel cells (HT‐PEMFCs) is regarded as a promising energy conversion system owing to simplified water management and enhanced tolerance to fuel impurities. However, phosphoric acid (PA) leaching remains a critical issue, diminishing energy density and durability, posing significant obstacle to the commercial development of HT‐PEMFCs. To address this, composite membranes incorporating the carboxylic acid‐modified polymer of intrinsic microporosity (cPIM‐1) are designed as framework polymer, blended with polyvinylpyrrolidone (PVP) for HT‐PEMFCs. The Lewis acid‐base interactions between cPIM‐1 and PVP created an extensive hydrogen‐bonding network, improving membrane compatibility. The optimized microporous structure and multiple anchoring sites gave rise to “domain‐limited” PA clusters, enhancing the capillary effect. Simultaneously, improved hydrophobicity synergistically optimizes catalytic interface, promoting continuous and stable proton transfer. The HT‐PEMFCs based on PVP/cPIM‐1 composite membrane achieved a peak power density of 1090.0 mW cm^−2^ at 160 °C, representing a 152% improvement compared to PVP/PES membrane. Additionally, it demonstrated excellent durability, with a voltage decay of 0.058 mV h^−1^ over 210 h of accelerated stress test corresponds to more than 5000 h of constant current density durability test. This study presents a promising strategy for the development of high‐performance and durable novel membranes in various energy conversion systems.

## Graphical abstract

A novel composite proton exchange membrane (PEM) design that leverages carboxylic acid‐functionalized polymers of intrinsic microporosity (cPIM‐1) and polyvinylpyrrolidone (PVP). Harnessing Lewis acid‐base interactions enables the development of a synergistic microporous structure that confines phosphoric acid clusters, enhancing proton conductivity and durability. This work addresses critical challenges in PEM development, while proposing a solutionfor the design of next‐generation membranes.

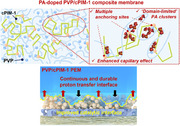

## 1 Introduction

Proton exchange membrane fuel cells (PEMFCs) have garnered considerable attention for their zero emissions and high energy conversion efficiency.^[^ ^1^, ^2^ ^]^ High‐temperature proton exchange membrane fuel cells (HT‐PEMFCs), operating in the range of 100–200 °C,^[^ ^3^ ^]^ hold significant promise for practical applications.^[^ ^4^ ^]^ Polybenzimidazole (PBI) is the predominant and most developed membrane material for HT‐PEMFCs due to its exceptional chemical and thermal stability.^[^ ^5^ ^]^ When doped with phosphoric acid (PA), the membrane's proton conductivity is significantly enhanced due to the formation of a hydrogen‐bonding network that facilitates effective proton transfer. However, in long‐term operations at high temperatures and at elevated acid doping levels (ADLs), the weakened van der Waals forces between PBI chains can cause various issues such as creep, detachment, PA loss, and mechanical degradation.^[^ ^6^, ^7^ ^]^ Cross‐linking is widely recognized for enhancing the mechanical properties of membranes and mitigating structural degradation.^[^ ^8^, ^9^ ^]^ However, this approach also introduces a trade‐off, as the occupation of PA binding sites can lead to a reduction in proton conductivity.^[^ ^10^ ^]^ Zhang et al. developed a gel‐state PBI membrane with a 3D layered structure, incorporating a dual cross‐linked phosphate bridge to facilitate proton conduction.^[^ ^11^ ^]^ This structure not only preserves high proton conductivity but also effectively reduces PA loss while improving creep resistance at temperatures exceeding 200 °C. Strengthening PA interactions with membrane molecules represents another viable strategy to address these challenges.^[^ ^12^, ^13^, ^14^ ^]^ Lee et al. utilized an in situ sol‐gel process to fabricate a composite membrane where the self‐assembled network cerium hydrogen phosphate (CeHP) significantly enhanced proton transport and extended the maximum operating temperature of HT‐PEMFCs to 250–300 °C.^[^ ^15^ ^]^ Nevertheless, the limited solution processability and high production costs of PBI and complex modification strategies still hinder its commercial viability.

Polyvinylpyrrolidone (PVP) features highly polar amide groups in its side chain, making it an excellent proton acceptor through hydrogen bonding.^[^ ^16^ ^]^ Due to its low toxicity, low manufacturing cost, and excellent processability, PVP‐based composite membranes have been widely studied for HT‐PEMFCs. However, the strong hydrophilicity and poor membrane‐forming properties of PVP result in excessive swelling of the membrane at high temperatures and under acidic conditions, rendering it unsuitable as a standalone PEM.^[^ ^17^ ^]^ Recent studies have shown that composite membranes prepared by blending PVP with engineered thermoplastic materials (as framework polymers), such as poly(vinylidene fluoride) (PVDF), poly(ether sulfone) (PES) and poly(etherketone‐calcinedoxin) (PEK‐c), can offer a degree of mechanical stability while maintaining proton conductivity in HT‐PEMFCs.^[^ ^18^, ^19^, ^20^ ^]^ The interaction between PVP and PVDF substantially enhances the compatibility of the composite membrane, however the ADL is limited to 2.7 due to the occupation of proton binding sites, leading to reduced performance.^[^ ^21^ ^]^ Further research on a PVP/PES composite membrane demonstrated negligible interaction between the two polymers. This absence of interaction allowed for a higher ADL (>5.9).^[^ ^22^ ^]^ While combining PA with side‐chain functional groups can enhance proton conductivity, it also introduces challenges. The “electrochemical pump” effect, where electrochemical reactions at the cathode generate water and create chemical potential gradients, without effective countermeasures, this gradient can cause PA to migrate from the membrane, resulting in performance degradation.^[^ ^23^ ^]^ Developing new framework polymers that restrict the mobility of free PA presents a more straightforward approach to mitigating PA leaching.

Polymers of intrinsic microporosity (PIMs) were first reported by Budd and McKeown in 2004.^[^ ^24^ ^]^ Due to their rigid structure and unique microporous properties, PIMs have gained increasing attention for applications in batteries,^[^ ^25^, ^26^ ^]^ fuel cells,^[^ ^27^ ^]^ and flow energy storage systems.^[^ ^28^, ^29^, ^30^ ^]^ In HT‐PEMFCs, PIM‐based proton exchange membranes have been reported to generate a siphoning effect,^[^ ^31^ ^]^ enhancing PA absorption, which significantly improves proton conductivity and enables operation across a broader temperature range. However, the durability of alkaline PIMs remains poor at high temperatures, due to excessive membrane swelling caused by their high ADL.^[^ ^32^ ^]^ Recently, functionalized‐PIMs have been reported as polymer binders for mitigation of the adsorption of PA on the catalyst surface, as the acid affinity can be adjusted by introducing various functional groups.^[^ ^33^ ^]^ With processability, intrinsic microporous and rigid structures, functionalized PIMs represent highly promising framework polymer capable of achieving PA management through interactions with other polymers.

**Figure 1:**
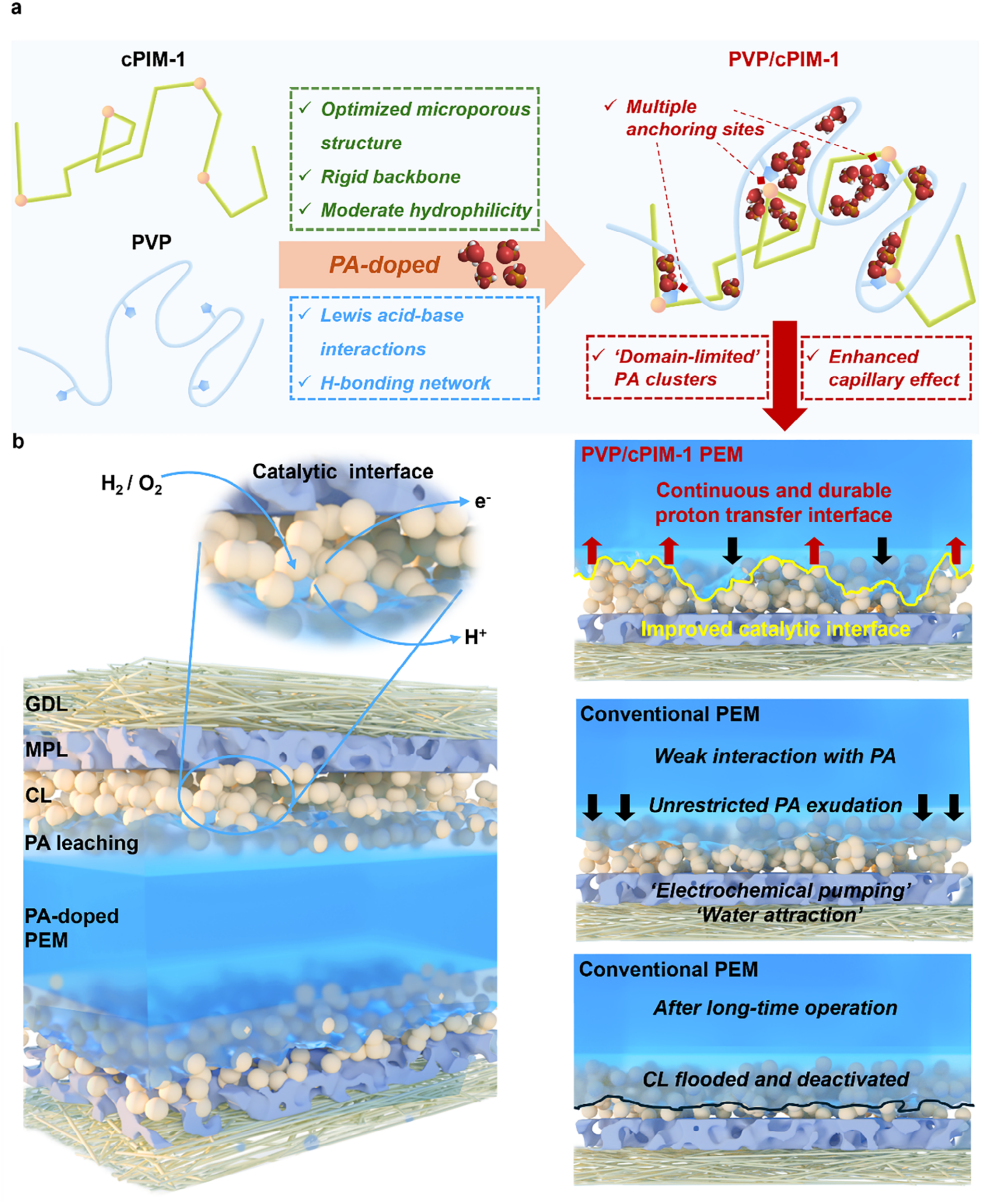
The design of PVP/cPIM‐1 composite membrane and membrane electrode assembly (MEA). a) The design concept of PVP/cPIM‐1 composite membrane. b) Schematic diagram of the MEA structure and the reactions of catalytic interface (GDL: Gas diffusion layer, MPL: Microporous layer, CL: Catalyst layer) (Left). PA exudation behavior of PVP/cPIM‐1and conventional PEM (Right).

Here, we designed composite membranes utilizing carboxylic acid‐functionalized PIM‐1 (cPIM‐1) as a framework polymer, blended with PVP for HT‐PEMFCs. The design of this composite membrane is motivated by the microporous structure of PIMs enhancing PA retention through capillary effects, while the rigid chain structure and excellent thermal stability provide a “structural guarantee” for long‐term stable operation. The amide groups and nitrogen‐containing heterocycles in the unit structure of PVP are considered generalized Lewis bases.^[^ ^34^ ^]^ The interactions between cPIM‐1 and PVP establish a stable and extensive hydrogen‐bonding network, enhancing compatibility.^[^ ^35^ ^]^ The optimized microporous structure synergizes with anchoring sites to create a broad array of domain‐limited free PA clusters, which mitigates polymer chain degradation enhancing the proton conductivity. The moderate hydrophobicity and interaction with PA optimize the catalytic interface, promoting continuous and stable proton transfer (**Figure** **1**). These properties contribute to the significantly improved durability of HT‐PEMFCs.

## 2 Results and Discussion

### 2.1 H‐Bonding Network and Improved Microporous Structure

**Figure 2:**
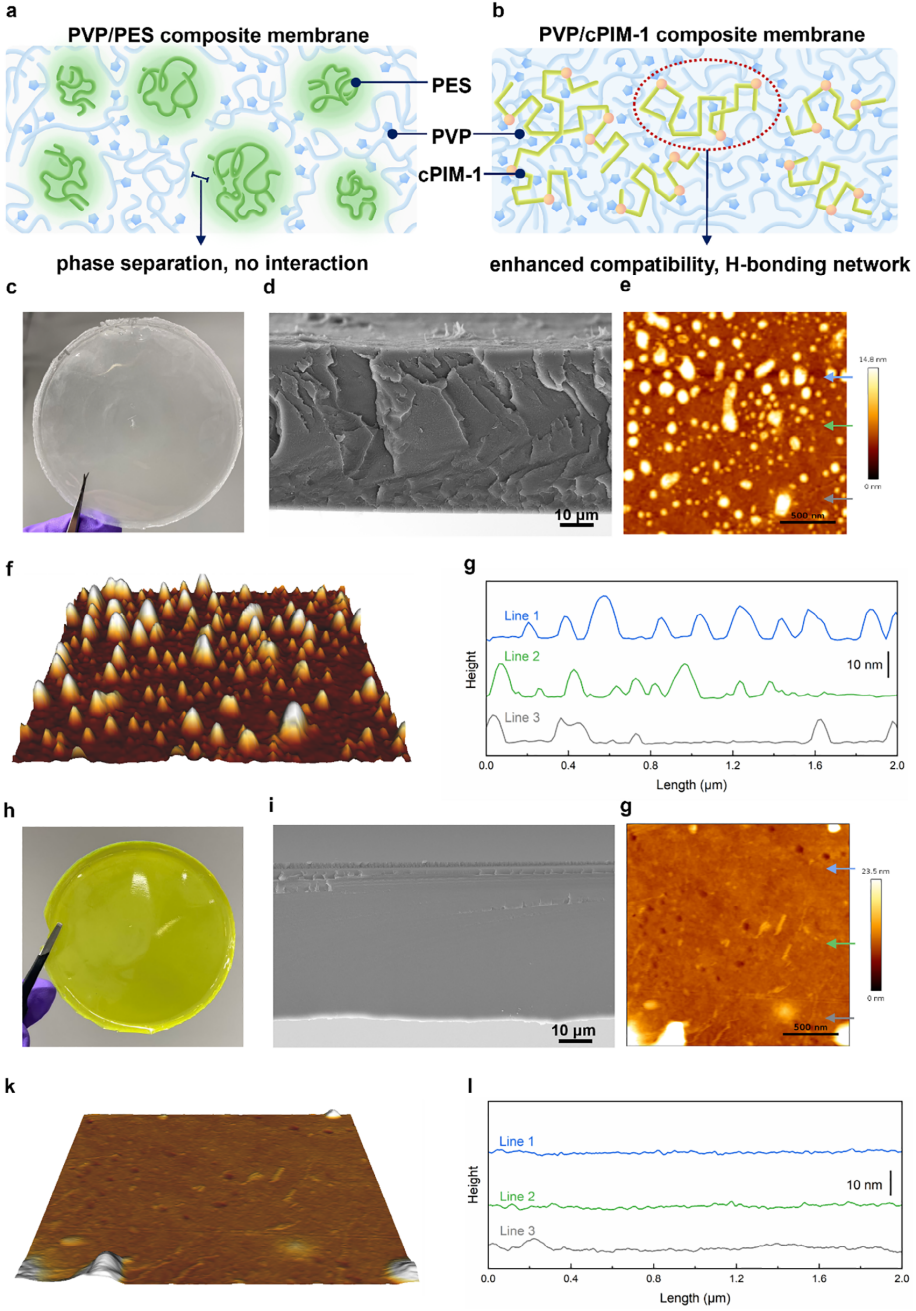
Interactions and morphology of composite membranes. Schematic diagram of a) PVP/PES and b) PVP/cPIM‐1. Digital photograph, SEM images (cross section), 2D and 3D AFM images (dry state, surface) and height profiles of c–g) 0.6 PVP/PES and h–l) 0.6 PVP/cPIM‐1.

PIM‐1 samples with different molecular weights were synthesized, and cPIM‐1 with different degrees of hydrolysis were obtained by high‐temperature acid hydrolysis (Figures S1 and S2, Supporting Information). The molecular structure and elemental composition of the polymers were confirmed by proton nuclear magnetic resonance (^1^H NMR), gel permeation chromatography (GPC) and elemental analysis, shown in Figure S2 and Tables S1 and S2 (Supporting Information). PVP/cPIM‐1 composite membranes were prepared by blending cPIM‐1 with PVP in different ratios (Table S3, Supporting Information). Compared to PVP/PES composite membranes, PVP/cPIM‐1 composite membranes, benefiting from inter‐chain interactions, are presumed to exhibit enhanced interfacial compatibility (**Figure** **2**a,b). The morphology of the PVP/cPIM‐1 composite membrane and the 0.6PVP/PES composite membrane were characterized by scanning electron microscopy (SEM) and atomic force microscopy (AFM) techniques. Both composite membranes with the same PVP loading, prepared by the solution casting method, exhibited homogeneous and transparent characteristics (Figure 2c,h; Figure S3, Supporting Information for other membranes). SEM images of the membrane cross sections (Figure 2d,i) revealed the rough texture of the 0.6PVP/PES membrane, while the 0.6PVP/cPIM‐1 membrane appeared very flat. Other composite membranes with different PVP/cPIM‐1 ratios displayed similar characteristics (Figure S4, Supporting Information). The AFM images of the PVP/PES membranes (Figure 2e–g) confirmed the presence of phase separation morphology, with the raised portions in the AFM representing self‐aggregation of polymer segments toward a state of lower free energy. However, this feature was not observed in the 0.6PVP/cPIM‐1 membrane or other cPIM‐1‐based composite membranes (Figure 2g–l; Figure S5, Supporting Information). In contrast, the PVP/cPIM‐1 composite membranes exhibited enhanced compatibility, which aligns with the anticipated outcomes of these framework polymers. To elucidate the interactions and confirm the presence of hydrogen bonding, Fourier‐transform infrared spectroscopy (FT‐IR), X‐ray diffraction (XRD), and proton nuclear magnetic (^1^H NMR) analyses (Figures S6–S8, Supporting Information) were conducted on the composite membranes. The amide carbonyl peak at 1654 cm⁻¹ in PVP broadens in the PVP/cPIM‐1, while the carboxylic acid carbonyl stretch at 1722 cm⁻¹ in cPIM‐1 shifts to higher wavenumbers, both changes associated with hydrogen bonding interactions.^[^ ^36^, ^37^ ^]^ In the XRD pattern, the diffraction peaks of cPIM‐1 are at ≈13.6° (d‐spacing = 6.5 Å) and 16.9° (d‐spacing = 5.2 Å), consistent with our previous work.^[^ ^38^ ^]^ In PVP/PES, the signals appearing at 22.3° represent the structures of PVP, and the sharp peak of PES appearing at 29.6°. These are likely due to the self‐aggregation and enrichment of certain PVP and PES domains, forming a partially phase‐separated structure.^[^ ^39^, ^40^ ^]^ However, the signals of cPIM‐1 and PVP are absent in the PVP/cPIM‐1 composite membranes pattern, this may be the result of hydrogen bonding, which strengthens inter‐segment interactions and increases the amorphous regions.^[^ ^41^ ^]^ The XRD results are consistent with the AFM images. Correspondingly, the carboxylate feature signal was also absent in the ^1^H NMR spectrum. Furthermore, molecular dynamics simulations of PVP/cPIM‐1 confirmed a widely distributed hydrogen bonding network within the binary polymer phases of PVP and cPIM‐1, and the chain model of PVP/cPIM‐1 reveals a more tightly packed structure, as illustrated in Figure S9 (Supporting Information).

**Figure 3:**
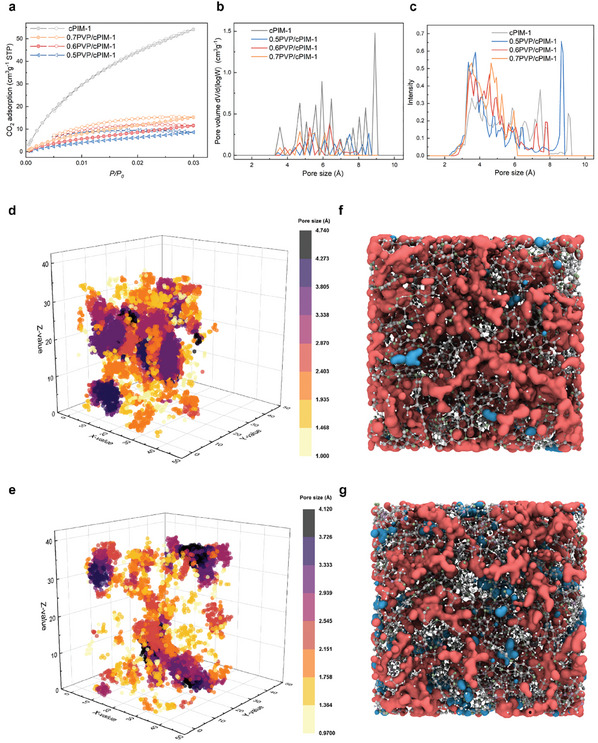
Porous structure characteristics. a) CO_2_ sorption isotherms at 273 K. b) Pore size distributions obtained from CO_2_ sorption isotherms calculated by density functional theory. c) Pore size distributions calculated based on molecular models of the membranes (3.3 Å probe). 3D pore size distribution and the amorphous unit cell of d,f) cPIM‐1 and e,g) 0.6PVP/cPIM‐1. Cyan indicates isolated micropores, while Cardinal red indicates interconnected micropores.

CO₂ adsorption was employed to characterize the microporous structure of cPIM‐1 and its composite membranes. The CO₂ adsorption of cPIM‐1 (**Figure** **3**a) aligned with that reported previously.^[^ ^42^ ^]^ The decrease in CO₂ adsorption in the composite membranes after the incorporation of PVP is probably due to the interaction between the two polymers, which reduces available adsorption sites and causes the polymer chains to pack more tightly, limiting the pore size. As determined by density functional theory (DFT) calculations, the pore size distribution (PSD) is presented in Figure 3b. In contrast to the wide distribution of cPIM‐1 (4–9 Å), the PSD of the PVP/cPIM‐1 composite membranes are more confined, narrowing to 3.3–6.5 Å. In Figure 3c, the PSD of PVP/cPIM‐1 obtained from simulated units using a 3.3 Å molecular probe were consistent with the experimental trends, which exhibited restricted pore sizes compared to cPIM‐1. Building on pore structure verification, a smaller molecular probe (0.85 Å) was employed to obtain a more detailed micropore distribution (Figure S10, Supporting Information). In Figure 3d,e, The 3D PSD within the 1–5 Å range reveals that the incorporation of PVP promotes a more dispersed and uniform micropore arrangement. This configuration can enhance the capillary effect on PA while facilitating a distributed continuous PA network within the membrane. The connectivity of the micropores was simulated as shown in Figure 3f,g and Figure S10b,c (Supporting Information), the proportion of non‐connected pores increased with higher PVP content, due to the tighter packing of polymer chain segments. The weak interactions among the free PA molecules are hypothesized to contribute to PA migration.^[^ ^43^ ^]^ This discrete pore structure can hinder the formation of large free PA clusters, while the proton‐conductive PVP segments forming the pore structure facilitate proton transport. Moreover, the capillary effect resulting from this confined microporous structure, coupled with the optimized distribution of discrete pores, offers conditions for significantly enhancing the PA uptake and retention.

### 2.2 “Domain‐Limited” Phosphoric Acid Clusters

**Figure 4:**
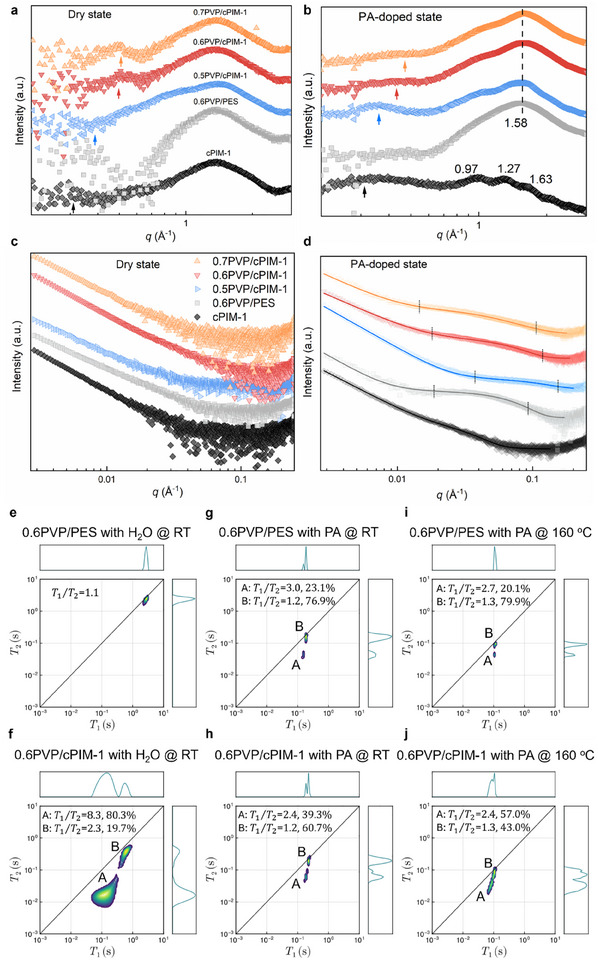
Characterization of PA clusters and interactions with polymers. Dry state and PA‐doped state a,b) WAXS and c,d) SAXS 1D plots of membrane samples. *T*_1_ − *T*_2_ relaxation correlation 2D plots of 0.6PVP/PES and 0.6PVP/cPIM‐1 for e,f) water, g,h) PA at room temperature and i,j) PA at 160 °C.

The hydrogen‐bond network and the restricted distribution of micropores facilitate the formation of a broadly distributed, confined cluster structures of PA molecules. To confirm this structure, small‐angle X‐ray scattering (SAXS) and wide‐angle X‐ray scattering (WAXS) were performed on both dry and PA‐doped membrane samples. All samples display an isotropic amorphous structure, as shown in Figure S11 (Supporting Information). The dry state WAXS (**Figure** **4**a) reveals that all cPIM‐1 based membrane samples exhibit distinct signals within a momentum transfer (*q*) range of 0.32–0.52 Å⁻¹, which is probably indicative of their porous structure.^[^ ^44^ ^]^ As the PVP content increases, the associated correlation length shows a tendency to decrease, aligning with the findings from our previous PSD simulations. The fluctuating signals observed in the PVP/PES sample within this range can be attributed to its localized phase separation. After PA‐doping (Figure 4b), the signal profile of the cPIM‐1 based membrane samples shows minimal variation, indicating that the rigid structure of cPIM‐1 effectively stabilizes the porous structure. Notably, the signal broadening observed in the PVP/cPIM‐1 samples suggests a high level of polydispersity at this correlation length. The fluctuations observed in 0.6PVP/PES disappear, possibly due to the swelling of polymer occupying the phase‐separated region. The PA‐doped cPIM‐1 sample retains characteristic peaks identical to those in dry state (the low peak signal intensity in the figure may be due to the background noise, the enlarged figure is shown in Figure S12a, Supporting Information), specifically at 0.97, 1.27, and 1.63 Å.^−1[^ ^45^ ^]^ This phenomenon can be attributed to the limited absorption of PA by cPIM‐1 (Table S4, Supporting Information), which results in no significant interaction and thus preserves its intrinsic peaks. The signal observed at 1.58 Å^−1^ in both dry state and PA‐doped state composite membrane samples is characteristic of PVP, consistent with the XRD results. The PVP/cPIM‐1 samples exhibit a new shoulder peak ≈1.0 Å⁻¹, probably attributed to the PA clusters within the porous structure, with a corresponding correlation length of ≈6.2 Å. The signal ≈0.4 Å⁻¹ in PVP/PES sample suggests that PA is concentrated in the phase separation structure, with a correlation length of ≈10 Å. The AFM characterization of the PA‐doped samples is presented in Figure S13 (Supporting Information). The PVP/PES composite membrane retains its morphology as observed in the dry state. The phase image distinctly reveals the characteristic aggregation domains of PVP and PES, along with the concentrated distribution of PA within the phase separation structure. The phase images of the PVP/cPIM‐1 composite membranes exhibit more uniform morphology with significantly reduced phase contrast, indicating a homogeneous distribution of PA within the membranes.

The dry state SAXS spectra (Figure 4c) reveals that the dry 0.6PVP/PES membrane exhibits a more gradual intensity decay in the low *q* region (q < 0.01 Å⁻¹) compared to the cPIM‐1‐based membranes, suggesting the presence of large‐scale (>10 nm) aggregates or interfacial inhomogeneities. This result is in agreement with our AFM findings. The PA‐doped state shows scattering signals distinct from those in the dry state within the *q* range of 0.01–0.1 Å⁻¹, likely due to the varying distributions of PA clusters within the samples. The Guinier approximation was applied to fit the SAXS data of the membrane samples using SASfit software.^[^ ^46^, ^47^ ^]^ Highly accurate fitting curves were obtained for all samples, except for the 0.7PVP/cPIM‐1 composite membrane. This discrepancy may be due to the more complex PA cluster structures and distribution in 0.7PVP/cPIM‐1 samples. For this sample, a polydisperse system of spherical particles with a log‐normal distribution was employed to achieve more accurate fitting results.^[^ ^48^ ^]^ Detailed fitting information can be found in Table S5 (Supporting Information). The radius of gyration (*R*_α_, in Guinier approximation) and fractal dimension (α) are used to describe the size and distribution of the scatterers. The cPIM‐1 sample still exhibited PA‐related scatterers, ≈5.82 nm in size. The α value of these scatterers is 3.29, indicating a relatively concentrated distribution of PA within the membrane. This may be due to the adsorption of PA by the intrinsic micropores, which leads to aggregate behavior and occupies most of the larger pore sizes. With the addition of PVP, the *R*_α_ of the composite membrane samples decreased significantly, ranging from 1.5 to 3.0 nm (the size distribution of 0.7PVP/cPIM‐1 sample is 0.3–4.2 nm, Figure S12b, Supporting Information). Meanwhile, the *R*_α_ in the 0.6PVP/cPIM‐1 sample is 2.7 nm, much smaller than that in the 0.6PVP/PES composite membrane (27.9 nm). These demonstrate that the PA clusters in the PVP/cPIM‐1 composite membrane are “domain‐limited”. Notably, the sizes of these “domain‐limited” PA clusters are smaller than those of the ion clusters or ion catchers structure reported in previous studies.^[^ ^49^, ^50^ ^]^ The gradual decrease in α values with increasing PVP content indicates that PA clusters become more dispersed within the membrane, as evidenced by the broadening of the signal range of 0.02–0.1 Å⁻¹. This change is attributed to the enhanced capillary effect of the confined micropores, while the interaction between PVP and cPIM‐1 “anchors” the free PA more effectively within the pores, promoting its widespread and stable distribution throughout the samples. In HT‐PEMFCs, proton transfer is widely regarded as being primarily governed by the vehicle mechanism.^[^ ^51^ ^]^ In PVP/cPIM‐1, the widely distributed “anchored PA” not only ensures a stable and high proton donor capacity but also benefits from the uniform microporous structure, which effectively reduces proton transport resistance.^[^ ^52^ ^]^ These synergistic effects collectively establish a highly efficient and stable proton transport pathway within the membrane.

To further elucidate the capillary interactions between PA and micropores, 2D *T*_1_‐*T*_2_ low‐field nuclear magnetic resonance (LF‐NMR) relaxation measurements were performed on PVP/cPIM‐1 and PVP/PES samples. This technique is well‐suited for assessing interfacial interactions of fluids in porous materials, as the *T*_1_/*T*_2_ ratio provides insights into the strength of the interface interaction between the probe molecule and the porous matrix.^[^ ^53^ ^]^ In this study, octane, water, and PA were selected as probe molecules and were tested to obtain *T*_1_/*T*_2_ values as controls (see Figure S14a–c, Supporting Information). The octane probe was used to assess the hydrophilicity of the samples. Higher *T*_1_/*T*_2_ values for PVP/cPIM‐1 indicated reduced hydrophilicity compared to PVP/PES, which was further corroborated by water contact angle measurements (see Figure S15, Supporting Information). In Figure 4e, using water as a probe molecule in the PVP/PES samples yielded a *T*_1_/*T*_2_ ratio of ≈1.1, and its peak shows a position similar to that of bulk water, suggesting that the water molecules are present as bulk water within the membrane. In contrast, the PVP/cPIM‐1 sample in Figure 4f shows two distinct peaks. The *T*_1_/*T*_2_ value of peak A is 8.3, and peak B has a *T*_1_/*T*_2_ value of 2.3. The strong interaction observed in peak A may be due to the strong confinement of water molecules within the porous structure. The 80.3% volume fraction of peak A indicates that this interaction is predominant, while the peak position of peak B suggests the presence of bulk water as well. This further verifies the widely distributed pore structure of the PVP/cPIM‐1 composite membranes. Using PA as the probe molecule at room temperature, both membrane samples exhibited two peaks as shown in Figure 4g,h. The position of peak B in both samples coincided with the peak of bulk PA, indicating the presence of free PA in both samples, as also observed for the case of water. Combining the findings from SAXS/WAXS, peak A in PVP/PES is assigned to PA located in the phase separation structure, while in PVP/cPIM‐1, it corresponds to PA within the pore structure. Peak A in PVP/PES exhibits a higher *T*_1_/*T*_2_ value of 3.0, indicating a stronger interaction for this material, as compared with PVP/cPIM‐1, between PA and the phase separation structure at room temperature. Meanwhile, the PVP/cPIM‐1 sample shows a higher volume fraction of 39.3%, suggesting that PA is mainly present in the porous structure, where capillary interactions play a significant role. After high‐temperature treatment (160 °C), as shown in Figure 4i, the *T*_1_/*T*_2_ ratio for peak A in PVP/PES decreased to 2.7, and the volume fraction reduced by 3.0%. This change may be due to the destabilization of the phase separation structure in the PVP/PES sample at high temperature, weakening the interaction with PA. However, as shown in Figure 4j, the *T*_1_/*T*_2_ ratio for peak A in PVP/cPIM‐1 remained unchanged, while the volume fraction increased to 57.0%. This suggests that, at high temperatures, the interaction between the microporous structure of PVP/cPIM‐1 and PA remained stable while the free PA tends to be more “drawn” into the pore structure. These results confirm the capillary effect of the microporous structure in PVP/cPIM‐1 on PA. In particular, the enhanced capillary action and the “anchoring” of free PA at high temperatures hold great potential for addressing the issue of PA leaching in HT‐PEMFCs.

### 2.3 High‐Temperature Fuel Cell Performance and Durability

**Figure 5:**
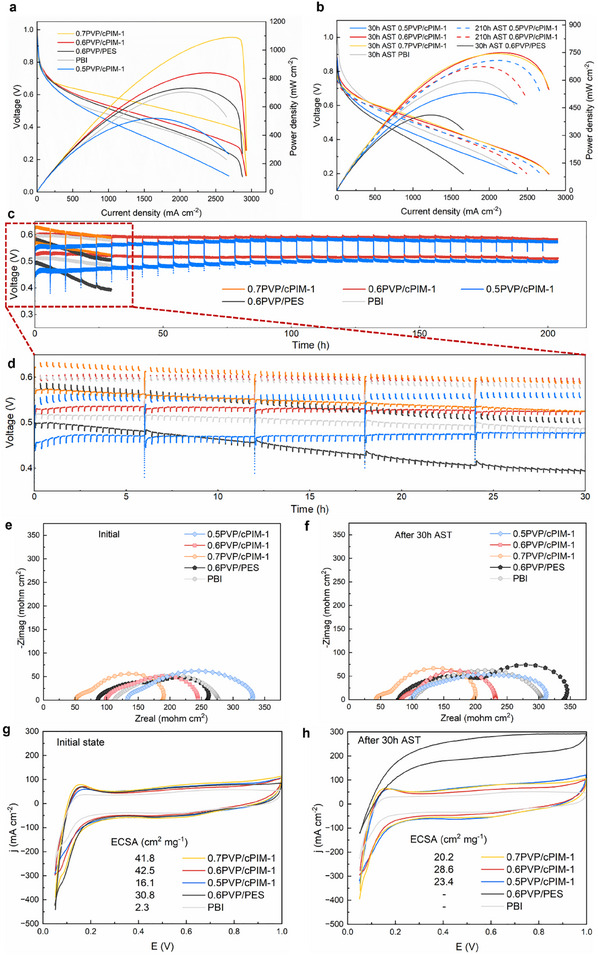
Fuel cell performance and electrochemical characterization. Polarization and power density curves of different MEAs: a) initial and b) after AST. Voltage degradation during AST: c) 210 h whole view, d) 30 h partial scaled‐up view. EIS fitted Nyquist curves of different MEAs at e) initial state and f) after 30 h AST (160 °C, anode: 100 mL min^−1^ hydrogen, 1 mg cm^−2^ Pt; Cathode: 100 mL min^−1^ oxygen, 1 mg cm^−2^ Pt). CV curves of different MEAs at g) initial state and h) after 30 h AST (160 °C, anode: 100 mL min^−1^ hydrogen, 1 mg cm^−2^ Pt; Cathode: 100 mL min^−1^ nitrogen, 1 mg cm^−2^ Pt).

The prepared PVP/cPIM‐1 composite membranes, along with PVP/PES and PBI membranes, were soaked in PA to prepare membrane electrode assembly (MEA) (see Table S4, Supporting Information for acid uptake, swelling rate, and other data). During the heating process, the open‐circuit voltage (OCV) was continuously monitored (Figure S16, Supporting Information). Notably, the OCV of the PVP/cPIM‐1 composite membrane consistently remained above 0.96 V, indicating its effective fuel barrier properties. The initial fluctuations in OCV were attributed to the equilibrium between the feed gas composition and variations in catalyst activity. Operating at 160 °C without back pressure or external humidification, as shown in **Figure** **5**a, the peak power density (PPD) of 0.6PVP/cPIM‐1 reached 839.4 mW cm⁻^2^. This represents a 14.6% increase to the MEA prepared with the PVP/PES membrane, which achieved a PPD of 732.1 mW cm⁻^2^ under identical PVP loading. Remarkably, 0.7PVP/cPIM‐1 achieved a PPD of 1090.0 mW cm⁻^2^, significantly surpassing both the PVP/PES and traditional PBI membranes, which reached only 704.8 mW cm⁻^2^. In addition, MEAs with lower catalyst loadings were also prepared for fuel cell performance testing, demonstrating superior performance compared to conventional PEMs (Figure S17, Supporting Information).

To assess membrane durability, an accelerated stress test (AST) program was implemented, specifically designed to intensify PA migration.^[^ ^54^ ^]^ As shown in Figure 5b, the 0.6PVP/cPIM‐1 membrane exhibited exceptional stability, maintaining PPDs of 750.0 mW cm⁻^2^ (90.0% retention) after 30 h of AST, and 673.9 mW cm⁻^2^ (80.3% retention) after 210 h of AST. The current density corresponding to the PPDs remained consistently high, within the range of 1800–2400 mA cm⁻^2^, suggests that the slight decrease in PPD is primarily attributed to an increase in charge transfer resistance rather than mass transfer limitations. Additionally, 0.5PVP/cPIM‐1 showed improved PPD over time, increasing from 533.0 mW cm⁻^2^ after 30 h AST to 707.3 mW cm⁻^2^ after 210 h AST, with the corresponding current density rising from 1800 mA cm⁻^2^ to 2100 mA cm⁻^2^. This enhancement is likely associated with its lower PVP/cPIM‐1 ratio, which results in stronger PA‐membrane interactions and well‐controlled PA leaching compared to other membranes. Before the initial PPD measurement, all MEAs underwent the same polarization curve activation process. However, at this stage, the PA distribution within the catalyst layer of 0.5PVP/cPIM‐1 was not yet optimized, leading to a lower initial PPD relative to other MEAs. After AST, PA gradual redistribution within the catalyst layer, improving the catalytic interface, which subsequently led to an increase in PPD. In contrast, both PVP/PES and PBI membranes exhibited a continuous decline in PPD, leading to eventual failure.

The voltage degradation during the AST is shown in Figure 5c,d. The voltage of the PVP/cPIM‐1 composite membrane remained stable across different current densities. Notably, the voltage of 0.5PVP/cPIM‐1 and 0.6PVP/cPIM‐1 increased gradually compared to their initial values, likely due to favorable redistribution of PA, which enhanced the reaction's three‐phase interface. For the 0.6PVP/cPIM‐1 membrane, the voltage steadily increased and remained stable during the first 30 h, before showing a slight decline after ≈150 h of testing. After 210 h of AST, the voltage degradation was remarkably low at just 0.058 mV h^−1^ at 0.6 A cm^−2^ and 0.086 mV h^−1^ at 1.0 A cm^−2^. To establish the correlation between the AST protocol and the constant current density durability test, the voltage degradation of 0.6 PVP/PES was evaluated under a constant current density of 0.6 A cm⁻^2^ (Figure S18, Supporting Information). The voltage loss induced by the AST was found to be ≈24 times that observed under the corresponding constant current density condition. This indicates that a voltage decay rate of 0.058 mV h⁻¹ over 210 h of AST corresponds to more than 5000 h of constant current density durability test. Compared to PVP/PES and PBI membranes, the PVP/cPIM‐1 composite membrane demonstrated superior durability. In addition, other MEAs using other cPIM‐1‐based composite membranes in this work were also tested (Figure S19, Supporting Information). Comparisons with other high‐performance membranes in the literature further highlight the overall advantages of PVP/cPIM‐1 (Table S6, Supporting Information). The corresponding equivalent circuit and the definition of parameters are shown in Figure S20 (Supporting Information). As shown in the initial Nyquist plot (Figure 5e), the semicircles of 0.6PVP/cPIM‐1 and 0.7PVP/cPIM‐1 exhibit a reduced diameter compared to 0.6PVP/PES and PBI in the medium‐frequency region, indicating enhanced electrode reaction kinetics and an improved membrane‐catalyst interface. Following 30 h of AST (Figure 5f), the semicircles of PVP/PES and PBI in the medium‐frequency region exhibited a significant increase, suggesting a deterioration of electrode kinetics due to uncontrolled PA exudation and a corresponding loss of catalytic active area. In contrast, 0.6PVP/cPIM‐1 and 0.7PVP/cPIM‐1 maintained nearly identical impedance characteristics, while 0.5PVP/cPIM‐1 showed a marked reduction in semicircle size, this downward trend has continued even after 210 h AST (Figure S21, Supporting Information). This decline is likely due to the redistribution of PA, which facilitated an enhancement in the effective catalytic interface. Thlectrochemically active surface area (ECSA) evolution also closely follows the trends observed in the Nyquist plots (Figure 5g,h). After 30 h of AST, the CV plots of PVP/PES and PBI exhibited no detectable hydrogen adsorption peaks. This absence was attributed to excessive PA leaching, flooding the catalyst surface and blocking the hydrogen adsorption sites.^[^ ^55^, ^56^ ^]^ A quantitative comparison of polarization resistance (*R_Pol_*)^[^ ^57^ ^]^ (Table S7, Supporting Information) further corroborates. In the initial state, both 0.6PVP/cPIM‐1 and 0.7PVP/cPIM‐1 exhibited substantially lower resistance than PBI and 0.6PVP/PES, whereas 0.5PVP/cPIM‐1 displayed higher resistance due to its well‐controlled PA leaching. After 30 h AST, the resistances of PBI and 0.6PVP/PES increased sharply due to uncontrolled PA leaching, leading to a reduced catalytic interface. In contrast, 0.6PVP/cPIM‐1 and 0.7PVP/cPIM‐1 remained remarkably stable, while 0.5PVP/cPIM‐1 exhibited a notable downward trend, further supporting the role of PA redistribution and appropriate hydrophobicity in optimising the catalytic interface. Even after 210 h AST, 0.5PVP/cPIM‐1 continued to show a decrease in, while 0.6PVP/cPIM‐1 exhibited only a marginal increase, confirming its stability under long‐term operational conditions. The ohmic resistance (*R_s_*), the charge transfer resistance of anode (*R*_*f* − *an*_) and the charge transfer resistance of cathode (*R*_*f* − *ca*_) of all samples obtained from EIS fitting (Table S8, Supporting Information). The *R_s_* of all the samples were decreased after AST, reflecting the membranes thinning after the long‐term operation. The initial *R*_*f* − *an*_ and *R*_*f* − *ca*_ of 0.6PVP/cPIM‐1 and 0.7PVP/cPIM‐1 exhibited significantly lower resistance compared to PBI and PVP/PES membranes, highlighting the exceptional charge transfer properties enabled by the optimized catalytic interface. Notably, this advantage became more pronounced after AST, with *R*_*f* − *an*_ and *R*_*f* − *ca*_ in 0.6PVP/cPIM‐1 and 0.7PVP/cPIM‐1 remaining nearly stable or exhibiting only a slight increase. In contrast, the *R*_*f* − *an*_ in PBI (29.7 to 59.8 mΩ cm^2^) and 0.6PVP/PES (69.7 to 121.0 mΩ cm^2^) nearly doubled after 30 h of AST, indicating significant degradation. The stability of *R*_*f* − *ca*_ in PBI is likely due to the attainment of a more uniform PA distribution, however, its resistance remained higher than that of the PVP/cPIM‐1 samples. Meanwhile, the *R*_*f* − *ca*_ of 0.6PVP/PES increased from 115.6 to 151.9 mΩ cm^2^, which may be attributed to a decline in the oxygen reduction reaction (ORR) rate at the cathode due to catalytic flooding. This observation aligns well with the calculated changes in *R_Pol_*. The resistance trends of 0.5PVP/cPIM‐1 were consistent with that of PPD and AST results. The distinct reduction in *R*_*f* − *ca*_ for PVP/cPIM‐1 is attributed to the favorable redistribution of PA and relatively enhanced hydrophobicity. The weakened interaction with water reduces the cathode's “water attraction” effect, thereby mitigating PA leaching.^[^ ^58^, ^59^ ^]^

### 2.4 PA Migration Behavior

**Figure 6:**
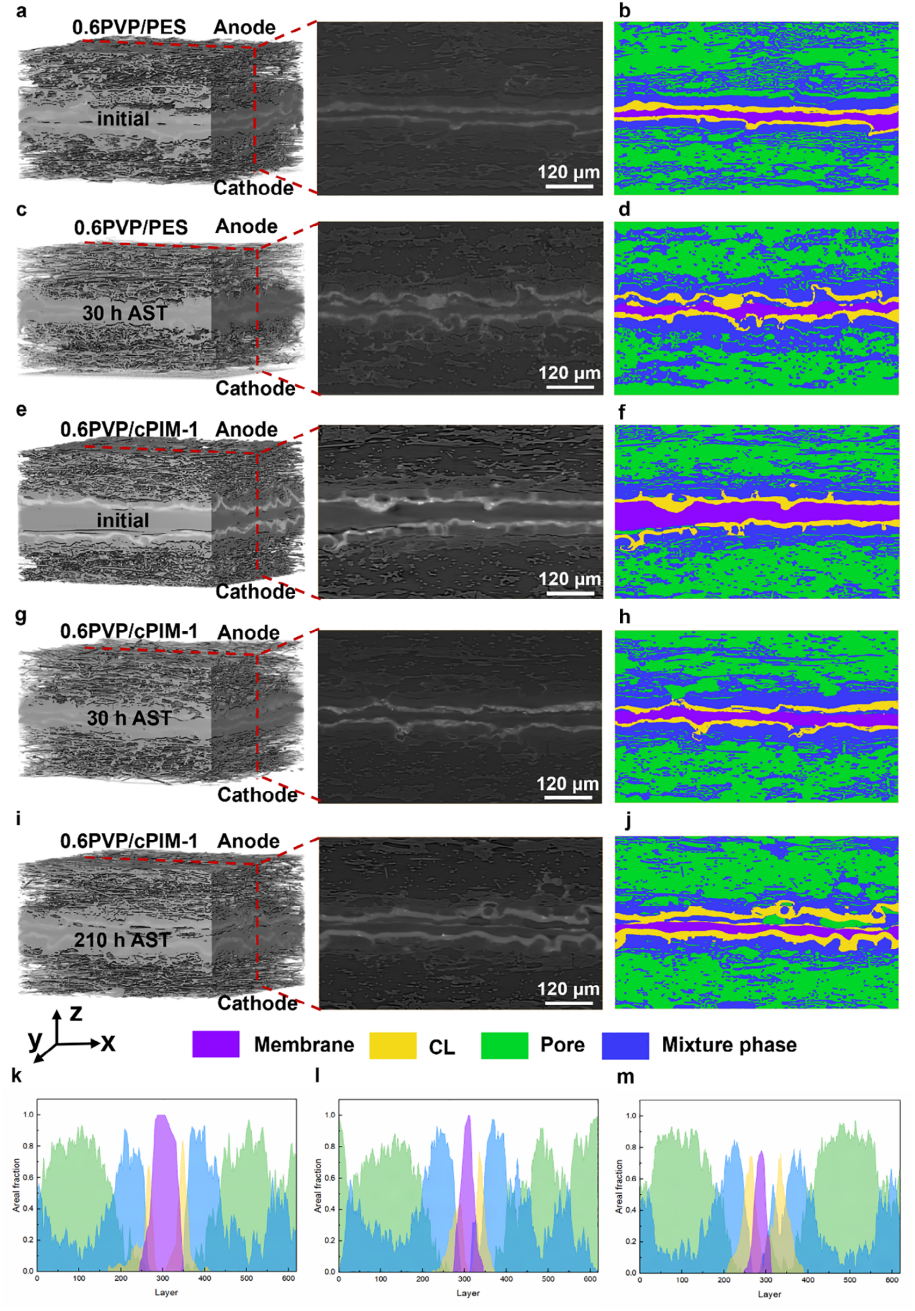
PA migration behavior in MEA. 3D X‐ray CT segmentation and the layer orthoslice of a,b) initial, c,d) 30 h AST 0.6PVP/PES and e,f) initial, g,h) 30 h AST, i,j) 210 h AST 0.6PVP/cPIM‐1. Slice‐by‐slice plots of area fraction in the Z‐direction of 0.6PVP/cPIM‐1 at k) initial, l) 30 h AST and m) 210 h AST state.

To investigate the differences in component migration and PA leaching under AST, characterized by the mechanical properties and PA retention capabilities of various membranes, X‐ray computed tomography (X‐ray CT) was employed. As shown in **Figure** **6**, panels a, c, e, g, and i represent the grayscale 3D and 2D cross sectional CT images of the MEAs for 0.6PVP/PES at 0 h, 0.6PVP/PES after 30 h of AST, 0.6PVP/cPIM‐1 at 0 h, 0.6PVP/cPIM‐1 after 30 h of AST, and 0.6PVP/cPIM‐1 after 210 h of AST, respectively. Figure 6b,d,f,h,j represents the segmentation of the corresponding components. Based on grayscale variations in CT images, the MEA components can be segmented into membrane, catalyst layer, pores, and other components, which collectively form a mixture phase. The mixture phase includes carbon fibers, carbon powder, PTFE binder, and leached PA. Although PA cannot be individually segmented, its leaching behavior can be inferred from changes in the mixture phase and pore structure.^[^ ^60^ ^]^ Based on Figure 6b,d, significant changes are observed in the MEA based on PVP/PES membranes before and after the 30 h AST. First, several areas of the membrane exhibit localized thinning, which can be a primary reason for the OCV decreases. Second, severe migration of the catalyst leads to a substantial reduction in adhesion to the membrane. Finally, as indicated by the blue regions representing the mixture phase, there is a noticeable expansion of the blue area from the membrane toward the sides, indicating a significant leaching of PA.

In contrast to the PVP/PES membranes, the MEA based on the 0.6PVP/cPIM‐1 membrane, as shown in Figure 6f,h, exhibits a notable reduction in thickness after 30 h AST without the occurrence of localized thinning. Additionally, the morphology of the catalyst remains well‐adhered to that of the membrane, and there is no significant expansion trend of the mixed phase. This can be attributed to the constrained migration behavior of PA, which prevents excessive leaching, as well as the optimized hydrophobicity of the membrane, which helps mitigate local water condensation in GDL, preserving its porous structure, thereby reducing gas transport resistance and further improving the catalytic interface. In contrast, 0.6PVP/PES and PBI exhibited significant CL migration and pore structure degradation, which contributed to their reduced catalytic efficiency. Moreover, as illustrated in Figure 6j, the mixture phase region after 210 h of AST not only did not exhibit further expansion but instead showed a contraction. This indicates that the rate of PA removal from the electrode during this stage has surpassed the rate of leaching from the membrane, demonstrating excellent control over PA leaching and contributing to its remarkable durability. The component fraction distribution maps of the MEA based on the 0.6PVP/cPIM‐1 membrane at different AST stages in the z‐direction, shown in Figure 6k–m, provide a more intuitive representation of component migration and distribution. Corresponding to Figure 6f,h,j, the peaks of the mixture phase in Figure 6k,l are similar near the catalyst, while the membrane peaks show some narrowing. However, after 210 h of AST, the mixed phase peak narrows, whereas the pore peak widens. This further indicates that the leaching of PA from the 0.6PVP/cPIM‐1 membrane is well‐regulated, thereby influencing its durability. The corresponding CT images and component distribution for the PBI membrane are shown in Figure S21 (Supporting Information).

This phenomenon can be further illustrated by calculating the phase fractions of the mixed phase (Table S9, Supporting Information). After 30 h AST, the phase fraction of the 0.6PVP/PES anode/cathode increased from 43.1%/40.4% to 45.0%/42.7%, a trend also observed in PBI. In contrast, the phase fractions of 0.6PVP/cPIM‐1 remained stable, shifting from 41.0%/38.1% to 41.8%/41.2%. After 200 h AST, the phase fractions decreased to 34.2% and 34.6%, likely due to the evaporation of PA and water caused by high temperatures and feed gas purging.^[^ ^61^, ^62^ ^]^ To assess the conductivity stability over time, the thickness of membrane samples was measured based on 3D X‐ray CT segmentation and the layer orthoslice at different AST time (30 and 210 h) to determine conductivity. As shown in Table S10 (Supporting Information), the conductivity of 0.6PVP/cPIM‐1 remains stable compared to 0.6PVP/PES sample.

## 3 Conclusion

In this study, cPIM‐1 was synthesized as framework polymer blended with PVP to fabricate PVP/cPIM‐1 composite membranes for HT‐PEMFCs. The acid‐base interactions between cPIM‐1 and PVP create an extensive hydrogen‐bonding network, enhancing the compatibility. The optimized microporous structure of the composite membrane, coupled with multiple anchoring sites for PA, gave rise to “domain‐limited” free PA clusters, thereby enhancing the capillary effect on PA. Compared to other HT‐PEMs based on PIM and PVP, the fuel cell performance and durability have improved significantly. The peak power density of the 0.7PVP/cPIM‐1 composite membrane reached 1090.0 mW cm^−2^ at 160 °C. Additionally, the improved hydrophobicity synergizes with these characteristics to improve the catalytic interface, enabling continuous and stable proton transfer. This addresses the challenge of performance decline due to excessive PA loss during prolonged fuel cell operation. After 210 h AST (corresponds to more than 5000 h of constant current density durability test.), the voltage decay was only 0.058 mV h^−1^. This study not only presents a novel strategy to address the challenge of performance decline due to excessive PA loss during prolonged operation in HT‐PEMFCs, but also provides valuable insights for the development of next‐generation membrane for different fuel cell systems (such as LT‐PEMFCs, anion exchange membrane (AEMFC) etc.). The microporous ion transport channels in PIM‐based composite membranes can be precisely tailored to accommodate diverse ion transport demands across varying operating conditions. We propose that the design of PIM‐based composite membranes developed in this study have significant potential for application across a wide range of energy conversion systems.

## 4 Experimental Section

### Materials

5,5′,6,6′‐Tetrahydroxy‐3,3,3′,3′‐tetramethyl‐1,1′‐spirobisindane (TTSBI, 97%) was purchased from Alfa Aesar. Tetrafluoroterephthalonitrile (TFTPN, >99%) was purchased from Fluorochem. Potassium carbonate (K_2_CO_3_, ≥99.5%), tetrahydrofuran (THF, ≥99.8%), sulfuric acid (H_2_SO_4_, ≥95%), and glacial acetic acid (≥99.7%) were purchased from Fisher Scientific. Polyethersulfone (PES, UltrasonÒ E7020P) was purchased from BASF Co., Germany. Polyvinylpyrrolidone (PVP, 1300 kDa), orthophosphoric acid (85%), polytetrafluoroethylene (PTFE) dispersion (60%), toluene (≥99.7%), N,N‐dimethylacetamide (DMAc, 99.8%), chloroform (≥99.8%), methanol (≥99.8%), 1,4‐dioxane (99.8%), acetone (≥99.5%), ethyl acetate (≥99.5%), hexane (≥97%), chloroform‐d (99.8 atom %D) and glass wool were purchased from Sigma‐Aldrich. Dimethylsulfoxide‐d_6_ (DMSO‑d_6_, D, 99.9%) was purchased from Cambridge Isotope Laboratories, Inc. ThePBI solution (1.1 IV, 18%) was purchased from PBI Performance Products, Inc., USA. Platinum on Carbon (XC‐72, 60%), electroconductive carbon black (Ketjenblack EC–300J) and Toray carbon paper (TGPH 090, 0.28 mm thickness) was purchased from The Fuel Cell Store. PTFE film (0.15 mm thickness) was purchased from Goodfellow. Kapton polyimide tape (50 mm wideness) was purchased from Youmile Co., China.

### Synthesis of PIM‐1

PIM‐1 synthesis was carried out using a high temperature procedure reported by Guiver et al.^[^ ^63^ ^]^ First, the TTSBI monomer purification is performed. TTSBI (40 g) was placed in a 3 L three‐neck, round‐bottom flask with a nitrogen flow input and reflux condenser. After the addition of 667 mL of ethyl acetate to the flask, the mixture was refluxed at 90 °C until complete dissolution of the monomer. Then, 667 mL of hexane was added to the flask and the mixture was allowed to stir for a further 10 min. After that, the flask was removed and given time to cool to room temperature. Once it had cooled down, it was placed in an ice bath for 3 h. The pure monomer was finally filtered out using a sintered glass funnel filter. The product was then placed in a vacuum for 24 h to dry. After the purification, TTSBI (17.02 g, 0.05 mol), TFTPN (10.00 g, 0.05 mol) and K_2_CO_3_ (20.73 g, 0.150 mol) were added in a 500 mL three‐neck round bottom flask. The flask was placed over a DrySyn heating block (Asynt, UK) over a hot plate magnetic stirrer (IKA, UK). The flask was connected to a nitrogen flow, an RZR mechanical stirrer (with digital rpm, torque, and time readings), and a coil condenser. Solvent mixture of DMAc (120 mL) and toluene (60 mL) were added to the flask, which equates to 20% excess solvent.^[^ ^64^, ^65^ ^]^ The mixture was heated to 160 °C while monitoring the temperature profile with a temperature probe. At the start of the reaction, mechanical stirring was set at 200 rpm; this speed was increased with the increase in the solution mixture's viscosity. Additional amounts of DMAc (20 mL) and toluene (10 mL) were added to reduce the overall viscosity of the solution at 27 and 36 min. The reaction was stopped after 45 min and quenched with an excess of methanol. The polymer was collected using a sintered filter under vacuum. For polymer purification, the recovered product was initially redissolved in chloroform (at a concentration of 5 g per 120 mL). The dissolved polymer was then re‐precipitated in methanol and collected via filtration once again. PIM‐1 was then refluxed in deionized (DI) water overnight and collected by vacuum filtration. The recovered polymer was immersed in 1,4‐dioxane for 15 min and washed with excess amounts of acetone and methanol. To remove any residues of other solvents, the polymer was immersed in methanol overnight. Finally, the filtered polymer was dried in a vacuum oven at 120 °C for few days until it dried completely.

### Synthesis and Purification of cPIM‐1

cPIM‐1 was synthesized by following the method proposed by the Smith Group.^[^ ^45^ ^]^ In a 1 L round‐bottom flask, 2.4 g of PIM‐1, 144 mL of DI water, 144 mL of sulfuric acid, and 48 mL of glacial acetic acid were added. The flask was put inside a Drysyn heating block on top of a hot plate magnetic stirrer and connected to a coil condenser. The hydrolysis reaction was performed at 150 °C for 24 h with magnetic stirring. Then the solution was allowed to cool down and diluted in 4 L of DI water. PIM‐1 was collected through vacuum filtration. The product was then refluxed in slightly acidic (few drops of sulfuric acid) DI water for 16 h. The final product was collected through vacuum filtration and then left overnight to dry in a 120 °C vacuum oven.

### Membrane Preparation

The cPIM‐1 membranes were also prepared by solution casting method. cPIM‐1 polymer was dissolved in DMAc solution to obtain 3 wt. % casting solution. After being filtered and cast in PTFE petri dishes, the membrane was obtained in 80 °C oven overnight, then in 130 °C vacuum oven for 4 h to remove the residual solvent. The 18 wt. % PBI solution was diluted to 3 wt. % with DMAc solution with mechanical stirring for 24 h at room temperature to obtain the PBI casting solution. The 0.18 g PVP and 0.12 g PES were each dissolved in 4 mL of DMAc respectively, then mixed together for 12 h to obtain 60 wt. % PVP/PES (0.6PVP/PES) casting solution. 0.18 g PVP and 0.12 g cPIM‐1 were each dissolved in 4 mL of DMAc respectively, at room temperature. The cPIM‐1 solution was filtered and mixed with PVP solution, then stirred for 12 h to obtain 60 wt. % PVP/cPIM‐1 (0.6PVP/cPIM‐1) casting solution. Membranes were prepared by casting method, in which the solutions were poured into Teflon Petri dishes and dried at 80 °C for 20 h, then under vacuum at 130 °C for 4 h to ensure the solvent was completely removed. The average thickness of the prepared membranes was 60 ± 5 µm.

### MEA Preparation

The prepared membrane was first soaked in 85 wt. % PA solution for 24 h at room temperature and then any excess PA was dried off the surface with filter paper. GDL of the electrode was prepared by spraying a microporous layer ink (formulated with 90 wt. % carbon black and 10 wt. % PTFE dispersion) on Toray carbon paper with a spray gun, after the carbon paper was completely dried, placed it in a muffle furnace and heated up to 300 °C for 3 h. The catalyst ink was obtained by blending the platinum/carbon catalyst (Pt content of 60 wt. %) with PTFE dispersion in water‐isopropanol solvent at a weight ratio of 4:1. The CL was prepared by spraying the catalyst ink onto the GDL until the platinum loading on the electrode reached 1 mg cm^−2^. A PTFE film of 0.15 mm thickness was used as the gas‐kit, and the gas‐kit was sealed with kapton polyimide tape where it contacted the membrane and the electrodes. The dried membrane was sandwiched between the two electrodes and hot‐pressed at 140 °C and 80 psi for 4.5 min to achieve an MEA with an active area of 5 cm^2^.

### Characterization of Polymers, Membranes, and MEA

The ^1^H NMR spectra on a Bruker Avance II 500 MHz instrument were recorded from 25 mg ml^−1^ of PIM‐1 in chloroform‐d solution and cPIM‐1 in DMSO‐d6, respectively. The number‐average molar mass (*M_n_*), weight‐average molar mass (*M_w_*), and dispersity (*Đ*) of PIM‐1 polymers were determined byGPC. Samples were prepared at a concentration of 1 mg ml^−1^ in chloroform (stabilized in ethanol). The samples were then examined using a triple detector Viscotek VE2001 system (Malvern Panalytical, UK) at a flow rate of 1 mL min^−1^ at 30 °C and an injection volume of 100 µL through two PL mixed‐B columns. The data were collected by OmniSec software.^[^ ^66^ ^]^ Elemental analysis was carried out by a Flash 2000 Organic Elemental Analyzer (Thermo Scientific, The Netherlands). Digital photography, SEM (FEI Nova Nano 450) and AFM (JPK Nanowizard; The Bruker Dimension Icon with ScanAsyst was used for PA‐doped membranes) were used to characterize the morphology and topology of the membranes. TGA of the materials and membranes was performed using the thermal analyzer (Q50, TA Instruments, USA). Materials and membranes were heated in the range of 25–600 °C at a heating rate of 10 °C min^−1^ in a nitrogen atmosphere at flow rate of 50 mL min^−1^. FT‐IR was conducted with a Nicolet iS 5 FT‐IR spectrometers (ThermoFisher Scientific). XRD measurements of membranes were performed on a D/MAX‐2500, and d‐spacing (*d*, nm) was calculated based on the Bragg equation:

(1) $$d=\frac{\lambda }{2\sin\theta }\;$$

where λ is the X‐ray wavelength (Cu Kα source, 1.54 Å), and θ is the X‐ray diffraction angle.

CO_2_ adsorption‐desorption measurements of the membranes were carried out on an ASAP 2460 (Micromeritics, USA) instrument at 273 K, respectively. The membranes (dry and PA‐doped states) were analyzed using SAXS and WAXS, proposing to acquire scattering profiles at 12.5 keV (0.689 Å) with a sample‐to‐detector distance of 2.87 m; this configuration allowed to investigate a Q‐range from 0.0025 to 3 Å^−1^ on the I22 SAXS Pilatus detector. The contact angle of the membranes was measured using a Krüss DSA 100 droplet shape analyzer with DI water. The diameter of the droplet needle was 0.5 mm. 2D *T_1_‐T_2_* relaxation correlation measurements were carried out with a  standard inversion recovery‐Carr‐Purcell‐Meiboom‐Gill (CPMG) sequence. The typical experimental error for all *T_1_‐T_2_* measurements was ≈3%. 16 recovery delays were used, from 1 ms to 10 s. The echo train of the CPMG sequence was composed varying in the range of 600 – 1600 echoes (dependent upon the sample) and acquired in a single shot with an echo spacing of 2τ = 0.5 ms, and an echo time of 250 µs. Each data set was acquired with 64 scans. A numerical inversion algorithm was used to process the experimental NMR data to obtain the 2D *T_1_‐T_2_* maps. NMR relaxation experiments were performed using a Magritek SpinSolve benchtop NMR spectrometer operating at a ^1^H frequency of 43 MHz. X‐ray CT measurements were performed on a diameter of 2 mm samples of the prepared MEA removed by laser micromachining. This method was a combination of laboratory X‐ray CT and machine‐learning segmentation as reported in previous work. All samples were scanned using a Zeiss Xradia 520 Versa (Carl Zeiss) X‐ray micro‐CT instrument equipped with a polychromatic source and a tube voltage of 30–160 kV.

PA uptake, volume swelling, area swelling, and ADL were given by the following equations,

(2) $$PA\;uptake=\frac{W_{wet}-W_{dry}}{W_{dry}}\;\times 100\%$$

(3) $$Volume\;swelling=\frac{V_{wet}-V_{dry}}{V_{dry}}\;\times 100\%$$

(4) $$Area\;swelling=\frac{S_{wet}-S_{dry}}{S_{dry}}\;\times 100\%$$

(5) $$ADL=\frac{\left(W_{wet}-W_{dry}\right)/M_{PA}}{\frac{W_{dry}\times i\;\%}{M_{cPIM-1}}+\frac{W_{dry}\times \left(1-i\right)\;\%}{M_{PVP}}}\;\times 100\%$$

where the mass, volume, and area of the membrane before and after PA soaking were notated as *W_dry_*, *W_wet_*, *V_dry_*, *V_wet_*, *S_dry_*, *S_wet_*, respectively. The *i* denotes the loading of cPIM‐1 in the composite membrane. The molecular weight of PA, cPIM‐1, and PVP were recorded as *M_PA_*, *M*_*cPIM* − 1_, and *M_PVP_*, respectively.

The proton conductivity was measured using electrochemical impedance spectroscopy (EIS) on a Gamry 5000E instrument at 160 °C without humidification. The EIS measurements were conducted under a constant DC current of 3.0 A, with a superimposed sinusoidal AC current of 0.1 A, the frequency range from 10000 to 0.1 Hz. The proton conductivity was calculated using the following equation,

(6) $$\sigma =\frac{L}{R\times A}\;$$

where σ is the proton conductivity (S cm^−1^), *L* is the membrane thickness (cm), *R* is the membrane resistance (Ω), and *A* is the membrane active area (cm^2^).

ECSA was determined through cyclic voltammetry (CV) measurements. The CV measurements conducted using a Gamry 5000E instrument at 160 °C under non‐humidified conditions. The anode and cathode were purged with 100 mL min^−1^ of H₂ and 100 mL min^−1^ of N₂, respectively, for 90 min before the measurements. CV scans were performed over a potential range of 0.05 to 1.0 V at a scan rate of 100 mV s^−1^. The ECSA was calculated by the following equation,

(7) $$ECSA=\frac{Q_{H}}{210\times m}\;$$

where *Q_H_* is the charge of the H_2_ adsorption (C), *m* is the catalyst loading (mg), 210 C cm^2^ is the standard charge required for a monolayer adsorption of H_2_ on Pt.

### Molecular Simulations

Model polymers were constructed using molecular dynamics simulations with the BIOVIA Materials Studio software. Each PVP and cPIMs polymer consisted of 10 and 14 structural units, respectively. In addition to pure cPIMs, mixtures of PVP and cPIMs were studied with weight ratios of 0.5, 0.6, and 0.7, each containing three cPIMs chains. The polymer construction process closely followed the methodology described in previous work,^[^ ^35^ ^]^ readers were encouraged to refer to the original article for further details. Briefly, the polymer chains were packed into a simulation box and the system undergoes an equilibration process that consists of a multistep annealing sequence. Temperature control was maintained using a Nosé‐Hoover thermostat with a coupling constant of 0.1 ps.^[^ ^67^ ^]^ The polymers were modeled using the COMPASSIII forcefield, and the equations of motion were integrated with a time step of 1 fs. A cutoff of 1.25 nm was employed for van der Waal interaction and the electrostatic interaction was calculated through Ewald sum. Structural properties were analyzed using PoreBlazer^[^ ^68^ ^]^ and ZEO++,^[^ ^69^ ^]^ where properties such as density, free volume, pore size distribution (including pore limiting diameter and largest cavity diameter), and pore percolation were evaluated and reported.

### Fuel Cell Performance Test

The prepared MEA was assembled into a fuel cell fixture (Scribner, effective area of 5 cm^2^) with a torque of 0.5 N m. The performance of the MEA was evaluated using an electrochemical workstation (Gamry, Reference 3000 with 30 K booster) at 160 °C unhumidified. All tests were performed using H_2_ and O_2_ in dry state at a flow rate of 100 mL min^−1^, respectively. The activation of the MEA was performed using the polarization curve activation method, cycling until the voltage profile stabilized. The polarization curve was obtained by discharging from OCV to 0.1 V with 0.05 A current increments. The AST performed chronopotentiometry at 0.6 A cm^−2^ (4 min) and 1.0 A cm^−2^ (16 min) alternately for 6 h, and the OCV was monitored during the testing. The EIS was measured before and after the chronopotentiometry, the equivalent circuit used for fitting the Nyquist curves is shown in Figure S19 (Supporting Information). All tests were analyzed using Gamry Echem Analyst software.

## Conflict of Interest

The authors declare no conflict of interest.

## Supporting information



Supporting Information

## Data Availability

The data that support the findings of this study are available from the corresponding author upon reasonable request.
