# Supplementary material for: Durable Proton Exchange Membrane Based on Polymers of Intrinsic Microporosity for Fuel Cells
Source: Adv Mater. 2025 Mar 27;37(19):2419534. doi: 10.1002/adma.202419534 (PMC12075903; doi:10.1002/adma.202419534)
Supplement: Supplementary file 1 — Supporting Information [file ADMA-37-2419534-s001.docx]

**Supplementary information**

**Durable Proton Exchange Membrane Based on Polymers of Intrinsic Microporosity for Fuel Cells**

Xiaochen Yang^1^, Zhiming Feng^1^, Mustafa Alshurafa^2^, Ming Yu^2,3^, Andrew B. Foster^2^, Heng Zhai^1^, Tianmu Yuan^1^, Yiheng Xiao^1^, Carmine D'agostino^1^, Ling Ai^1^, Maria Perez-Page^1^, Keenan Smith^4^, Fabrizia Foglia^4^, Adam Lovett^5^, Thomas S. Miller^5^, Jianuo Chen^1,5, ***^, Peter M. Budd^2, **^, Stuart M. Holmes^1, *^

*^1^Department of Chemical Engineering, The University of Manchester, Manchester, M13 9PL, United Kingdom*

*^2^Department of Chemistry, The University of Manchester, Manchester, M13 9PL, United Kingdom*

*^3^Department of Chemical Engineering, The University of Melbourne, Melbourne, VIC, 3010, Australia*

*^4^Department of Chemistry, University College London, London, WC1H 0AJ, United Kingdom*

*^5^Department of Chemical Engineering, University College London, London, WC1E 7JE, United Kingdom*

* Corresponding author: e-mail: stuart.holmes@manchester.ac.uk

** Corresponding author: e-mail: peter.budd@manchester.ac.uk

*** Corresponding author: e-mail: jianuo.chen@ucl.ac.uk

| **Supplementary Figure 1.** | Polymerization and functionalization process: a) Branched PIM-1 was produced by nucleophilic substitution polymerization of TTSBI and TFTPN via a modified high-temperature method. b) cPIM-1 is produced by the acid hydrolysis of PIM-1. |
| --- | --- |
| **Supplementary Figure 2.** | ^1^H NMR spectra of a) PIM-1 and cPIM-1, b) PIM-1a and cPIM-1a, c) PIM-1b and cPIM-1b. All PIM-1 and cPIM-1 samples were analyzed in CDCl_3_ and DMSO- d_6_, respectively. The serial numbers of the characteristic peaks in spectra correspond to those in Supplementary Figure 1 |
| **Supplementary Figure 3.** | Digital photographs of the membranes: a) PBI, b) cPIM-1, c) 0.5PVP/cPIM-1, d) 0.7PVP/cPIM-1. |
| **Supplementary Figure 4.** | Cross-section SEM images of membranes: a) PBI, b) cPIM-1, c) 0.5PVP/cPIM-1, d) 0.7PVP/cPIM-1 (Mag=20.00 KX). |
| **Supplementary Figure 5.** | Height profiles and 3D AFM images of membranes. a,b) PBI, c,d) cPIM-1, e,f) 0.5PVP/cPIM-1, g,h) 0.7PVP/cPIM-1. |
| **Supplementary Figure 6.** | FT-IR spectra of PVP, PES (polymers) and PIM-1, cPIM-1 0.6PVP/PES composite membrane and PVP/cPIM-1 composite membranes. |
| **Supplementary Figure 7.** | XRD patterns of membrane samples of cPIM-1, PVP/PES and PVP/cPIM-1. |
| **Supplementary Figure 8.** | ^1^H NMR spectra of PVP (polymer) and membrane samples of cPIM-1 and PVP/cPIM-1 in DMSO d_6_. |
| **Supplementary Figure 9.** | Chain model (left) and the hydrogen bonding distribution (right) calculated by molecular dynamics simulations in the unit cells of a) cPIM-1, b) 0.5PVP/cPIM-1, c) 0.6PVP/cPIM-1 and d) 0.7PVP/cPIM-1. Red: O, gray: C. blue: N, white: H. |
| **Supplementary Figure 10.** | a) Pore size distribution derived from molecular dynamic simulation based on Voronoi decomposition (0.85 Å molecular probe). 3D pore size distribution in the range of 0-5 Å (left) and the amorphous unit cell (right) of b) 0.5PVP/cPIM-1 and c) 0.7PVP/cPIM-1 membrane. Cyan indicates isolated micropores, while Cardinal red indicates interconnected micropores. |
| **Supplementary Figure 11.** | SAXS and WAXS 2D patterns in dry state and PA-doped state of a,b) 0.6PVP/PES, c,d), cPIM-1 e,f) 0.5PVP/cPIM-1 and g,h) 0.6PVP/cPIM-1 and i,j) 0.7PVP/cPIM-1. X-axis and Y-axis: pixels. |
| **Supplementary Figure 12.** | a) Dry-state WAXS enlarged view of cPIM-1 sample, b) the scatterers size distribution of 0.7PVP/cPIM-1 sample. |
| **Supplementary Figure 13.** | AFM Height and phase profiles of PA-doped membranes. a,b) 0.6PVP/PES, c,d) 0.5PVP/cPIM-1, e,f) 0.6PVP/cPIM-1, g,h) 0.7PVP/cPIM-1. |

| **Supplementary Figure 14.** | $T_{1}-T_{2}$ relaxation correlation 2D plots of a) water at room temperature, PA b) at room temperature, c) at 160 ^o^C, d,e) 0.6PVP/PES and 0.6PVP/cPIM-1 for octane at room temperature. |
| --- | --- |
| **Supplementary Figure 15.** | Digital photographs of water contact angles of a) 0.6PVP/PES, b) cPIM-1, c) PBI and d) 0.6PVP/cPIM-1. |
| **Supplementary Figure 16.** | OCV curves during fuel cell pre-heating (heating from room temperature and reaching 160 ^o^C at 1200s, then maintain 300s). |
| **Supplementary Figure 17.** | Polarization curves and power density curves of different MEAs with a) 0.3 mg cm^-2^ Pt loading and b) 0.5 mg cm^-2^ Pt loading, different Pt loading in c) 0.6PVP/cPIM-1, d) 0.7PVP/cPIM-1. |
| **Supplementary Figure 18.** | Constant current density durability test of 0.6PVP/PES. (160 ℃, anode: 100 mL min^-1^ hydrogen, 1 mg cm^-2^ Pt; Cathode: 100 mL min^-1^ oxygen, 1 mg cm^-2^ Pt). |
| **Supplementary Figure 19.** | HT-PEMFCs performance of cPIM-1 composite membranes with different hydrolysis times. a) polarization curves and power density curves, b) AST process, c,d) fitted EIS Nyquist curves before and after AST process. (160 ℃, anode: 100 mL min^-1^ hydrogen, 1 mg cm^-2^ Pt; Cathode: 100 mL min^-1^ oxygen, 1 mg cm^-2^ Pt). |
| **Supplementary Figure 20.** | The equivalent circuit, definition of parameters and EIS data fitting schematic. |
| **Supplementary Figure 21.** | EIS fitted Nyquist curves of 0.5PVP/cPIM-1 and 0.6PVP/cPIM-1 after 210h AST. (160 ℃, anode: 100 mL min^-1^ hydrogen, 1 mg cm^-2^ Pt; Cathode: 100 mL min^-1^ oxygen, 1 mg cm^-2^ Pt). |
| **Supplementary Figure 22.** | 3D X-ray CT segmentation and the layer orthoslice of a,b) initial, c,d) 30h AST PBI. Slice-by-slice plots of area fraction in the Z-direction of e,f) initial and 30h AST PBI and g,h) initial and 30h AST 0.6PVP/PES. |
| **Supplementary Table 1** | Details of PIM-1 synthesized from step-growth polymerizations and characterization results: Solvent, Polymer scale, Polymerization temperature, time, yield, branching, weight-average molar mass (Mw), number-average molar mass (Mn), dispersity (Đ) and intrinsic viscosity. |
| **Supplementary Table 2** | Elemental analyses of PIM-1 and cPIM-1, and conversion of -COOH calculations. |
| **Supplementary Table 3** | Composite membrane nomenclature and composition. |
| **Supplementary Table 4** | The acid uptake, proton conductivity, volume and area swelling, and ADL of membrane samples. |
| **Supplementary Table 5** | SAXS fitting results of PA-doped state membrane samples. |
| **Supplementary Table 6** | Comparison and summary of the fuel cell performance based on PVP-based and PIM-based leading membranes. |
| **Supplementary Table 7** | $R_{Pol}$ of different MEAs before and after AST obtained from equivalent circuits. |
| **Supplementary Table 8** | $R_{s}, R_{f-an} and R_{f-ca}$ of different MEAs before and after AST obtained from equivalent circuits. |
| **Supplementary Table 9** | GDL/MPL/PA phase fraction of different MEA samples. |
| **Supplementary Table 10** | Proton conductivity of different membrane samples at different stages. |
| **References** | (1-25) |

**
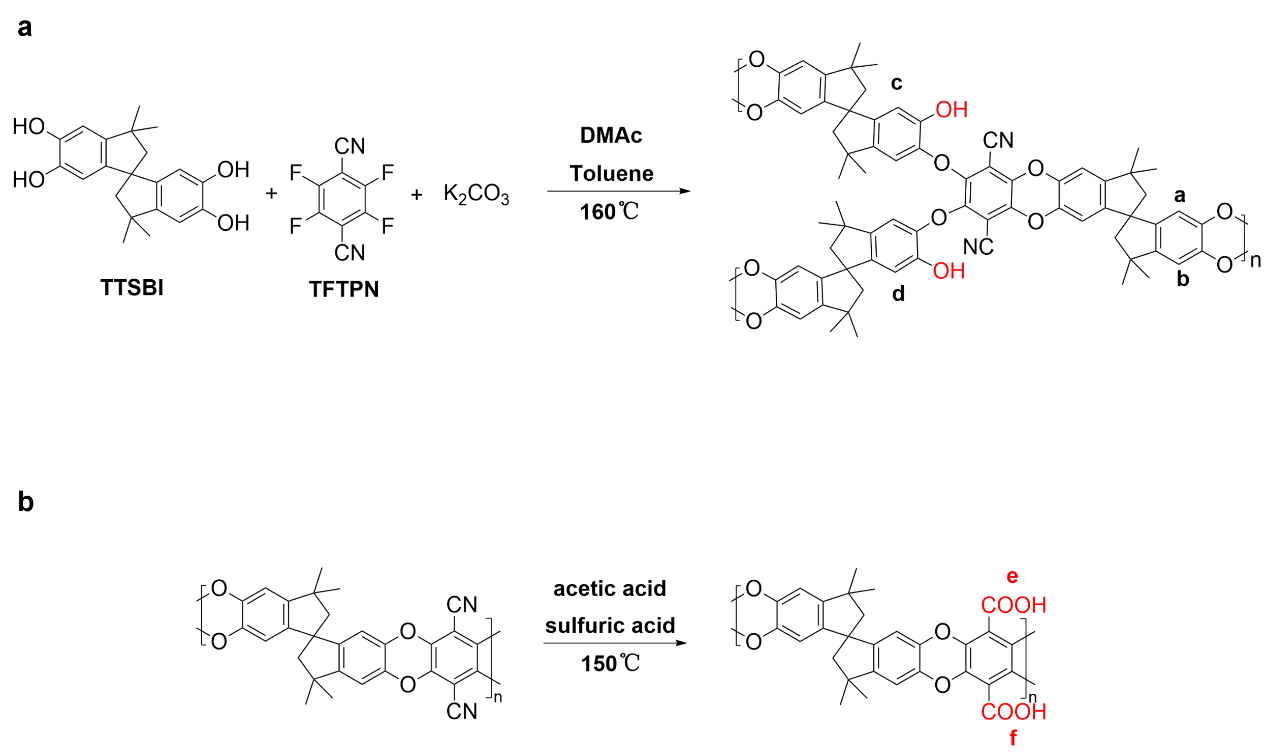
**

**Supplementary Figure 1.** Polymerization and functionalization process: a) Branched PIM-1 was produced by nucleophilic substitution polymerization of TTSBI and TFTPN via a modified high-temperature method. b) cPIM-1 is produced by the acid hydrolysis of PIM-1.

**
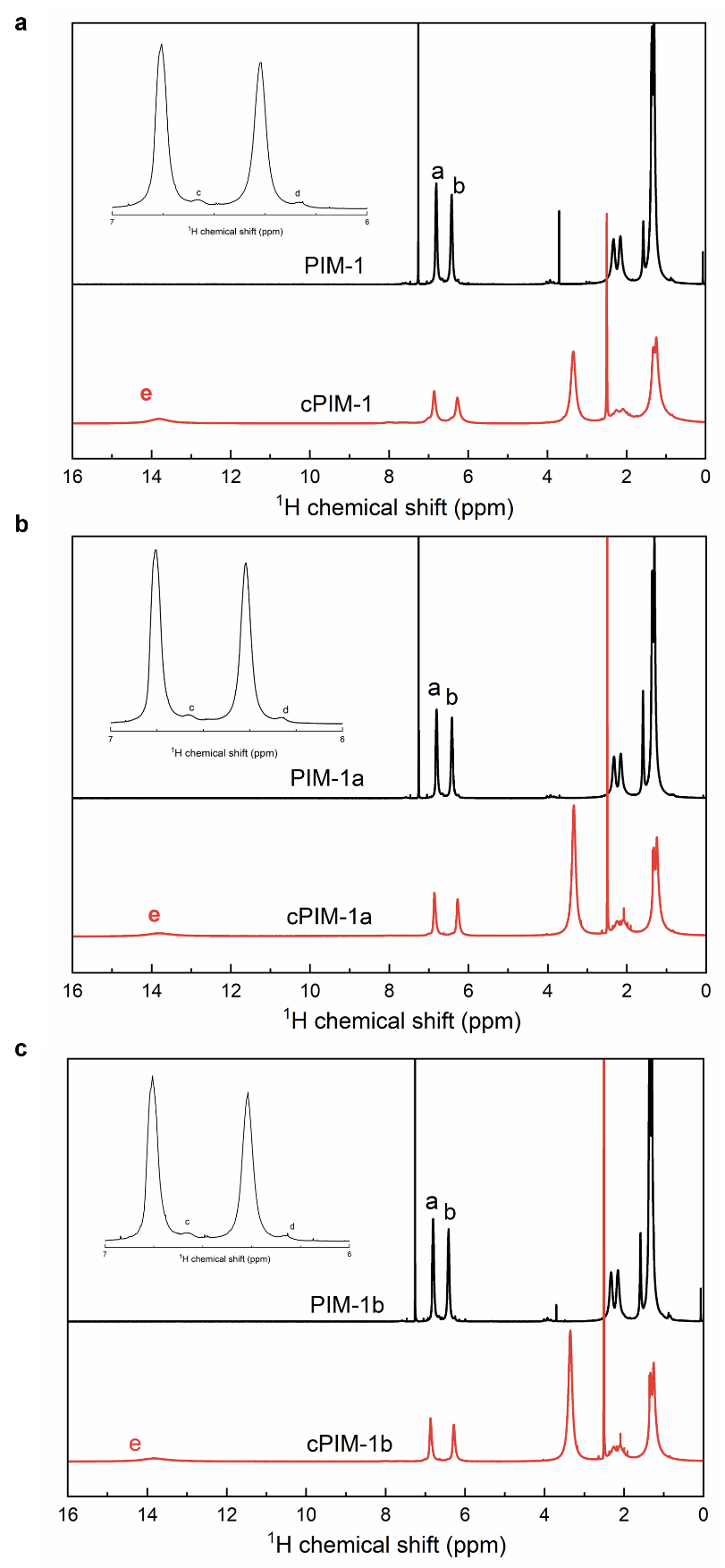
**

**Supplementary Figure 2.** ^1^H NMR spectra of a) PIM-1 and cPIM-1, b) PIM-1a and cPIM-1a, c) PIM-1b and cPIM-1b. All PIM-1 and cPIM-1 samples were analyzed in CDCl_3_ and DMSO-d_6_, respectively. The serial numbers of the characteristic peaks in spectra correspond to those in Supplementary Figure 1.


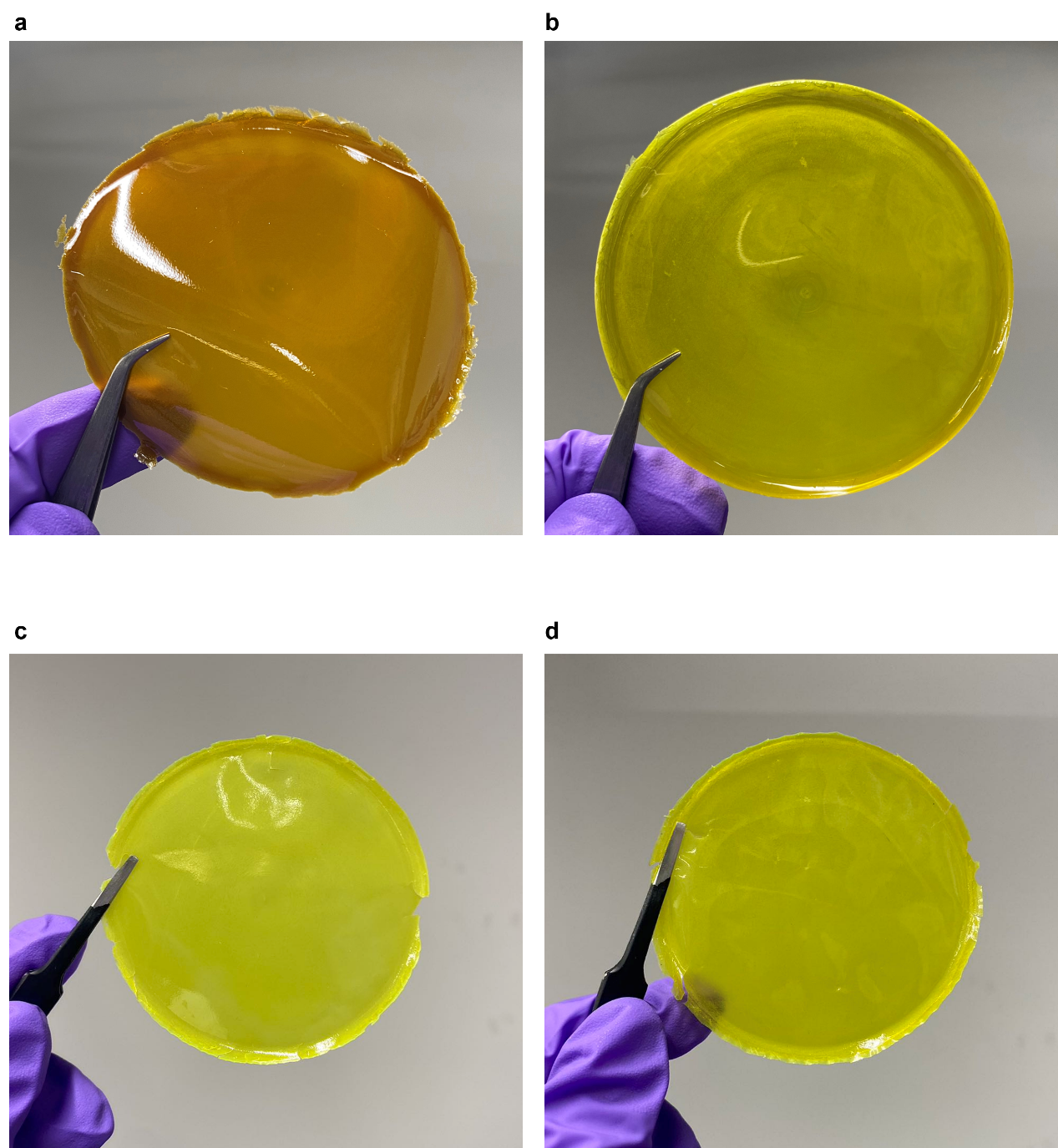


**Supplementary Figure 3.** Digital photographs of the membranes: a) PBI, b) cPIM-1, c) 0.5PVP/cPIM-1, d) 0.7PVP/cPIM-1.

^
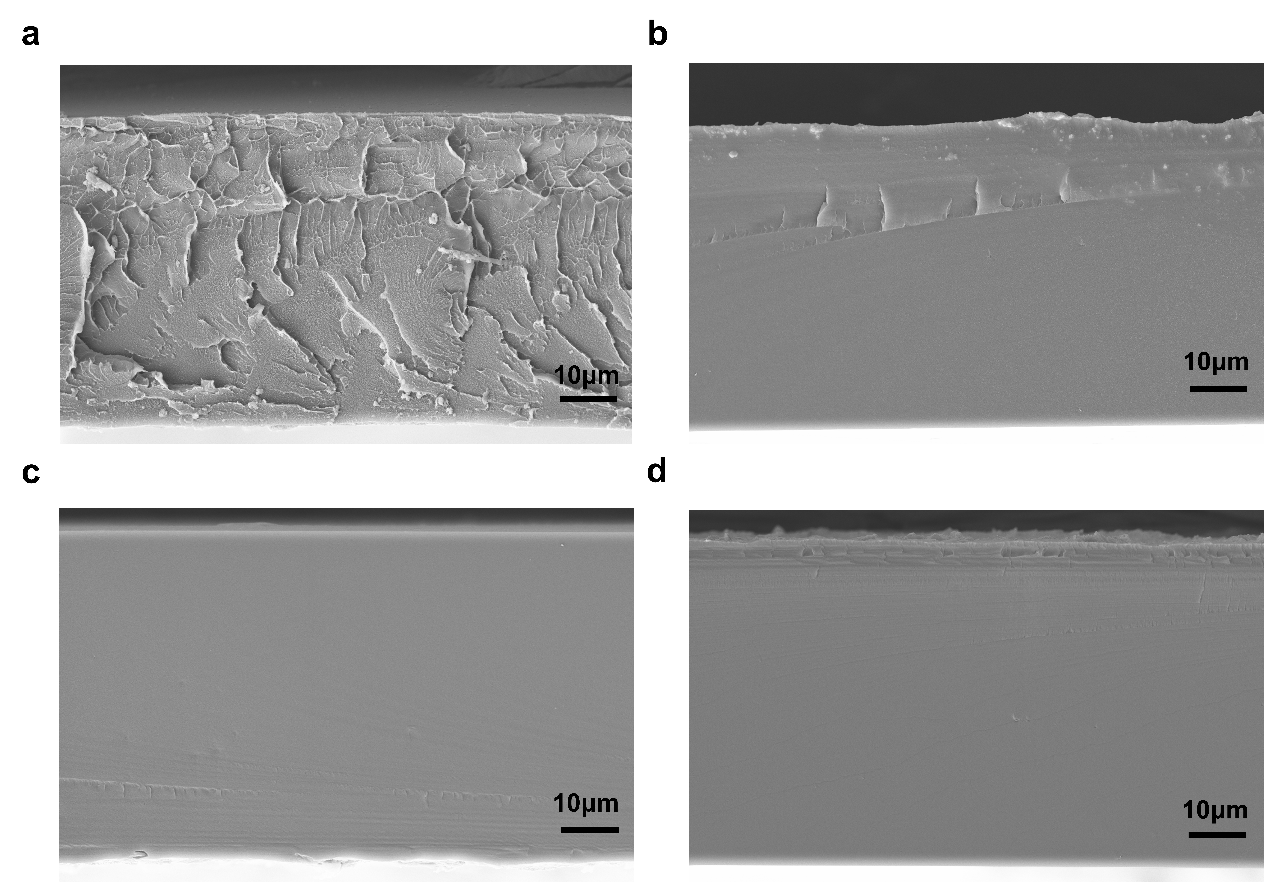
^

**Supplementary Figure 4.** Cross-section SEM images of membranes: a) PBI, b) cPIM-1, c) 0.5PVP/cPIM-1, d) 0.7PVP/cPIM-1 (Mag=20.00 KX).


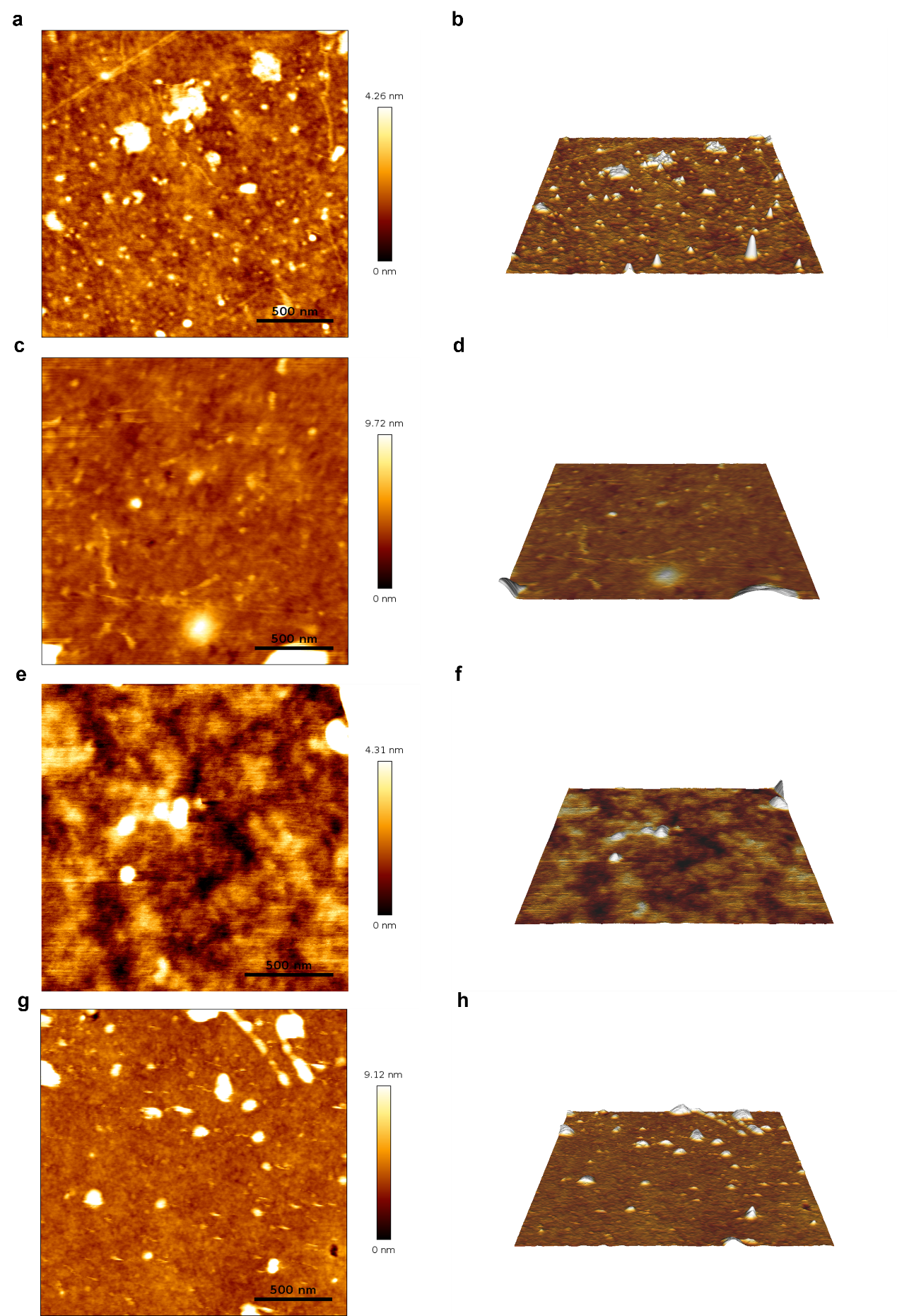


**Supplementary Figure 5.** Height profiles and 3D AFM images of membranes. a,b) PBI, c,d) cPIM-1, e,f) 0.5PVP/cPIM-1, g,h) 0.7PVP/cPIM-1.

**

**

**Supplementary Figure 6.** FT-IR spectra of PVP, PES (polymers) and PIM-1, cPIM-1 0.6PVP/PES composite membrane and PVP/cPIM-1 composite membranes.

**

**

**Supplementary Figure 7.** XRD patterns of membrane samples of cPIM-1, PVP/PES and PVP/cPIM-1.


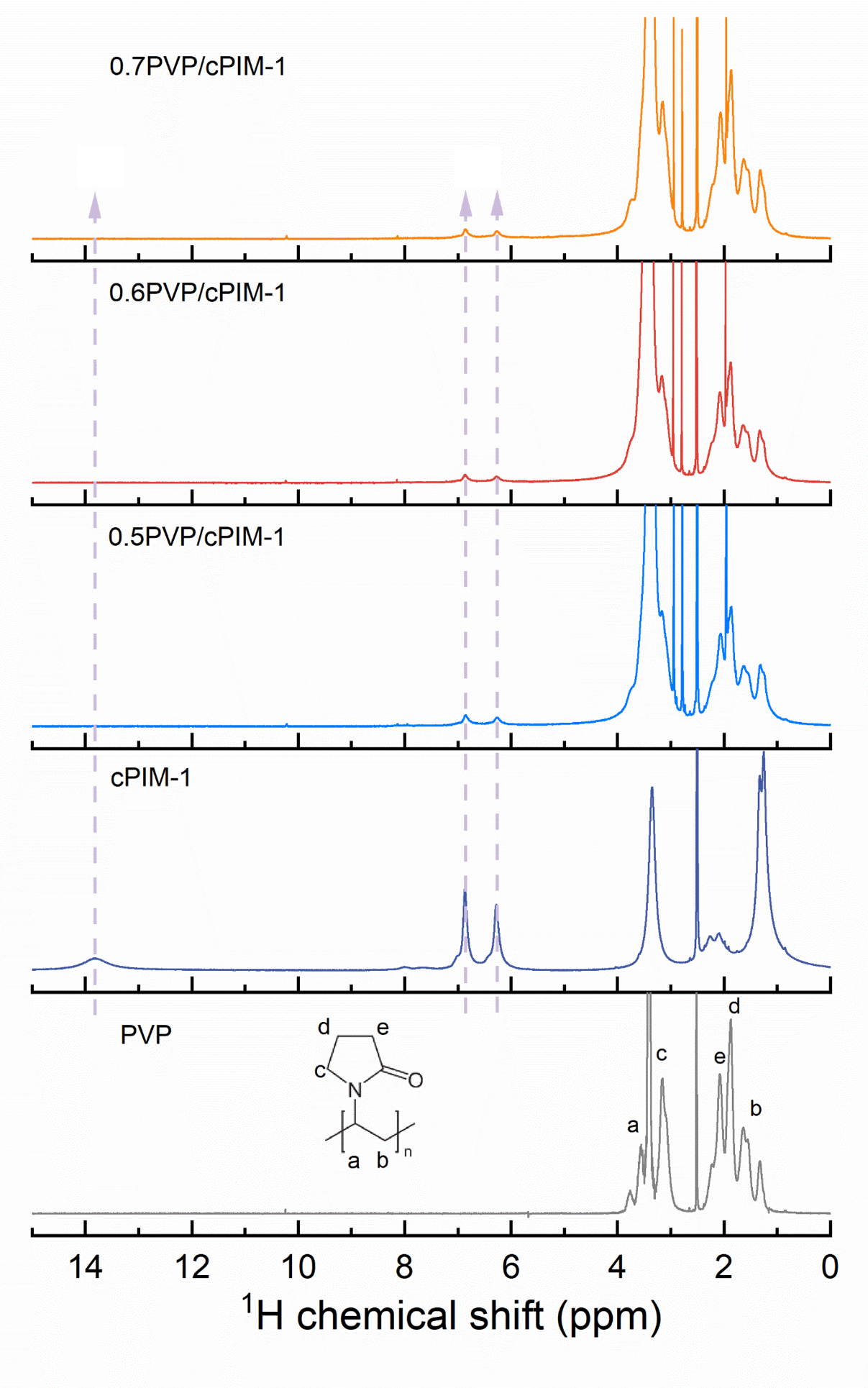


**Supplementary Figure 8.** ^1^H NMR spectra of PVP (polymer) and membrane samples of cPIM-1 and PVP/cPIM-1 in DMSO d_6_.


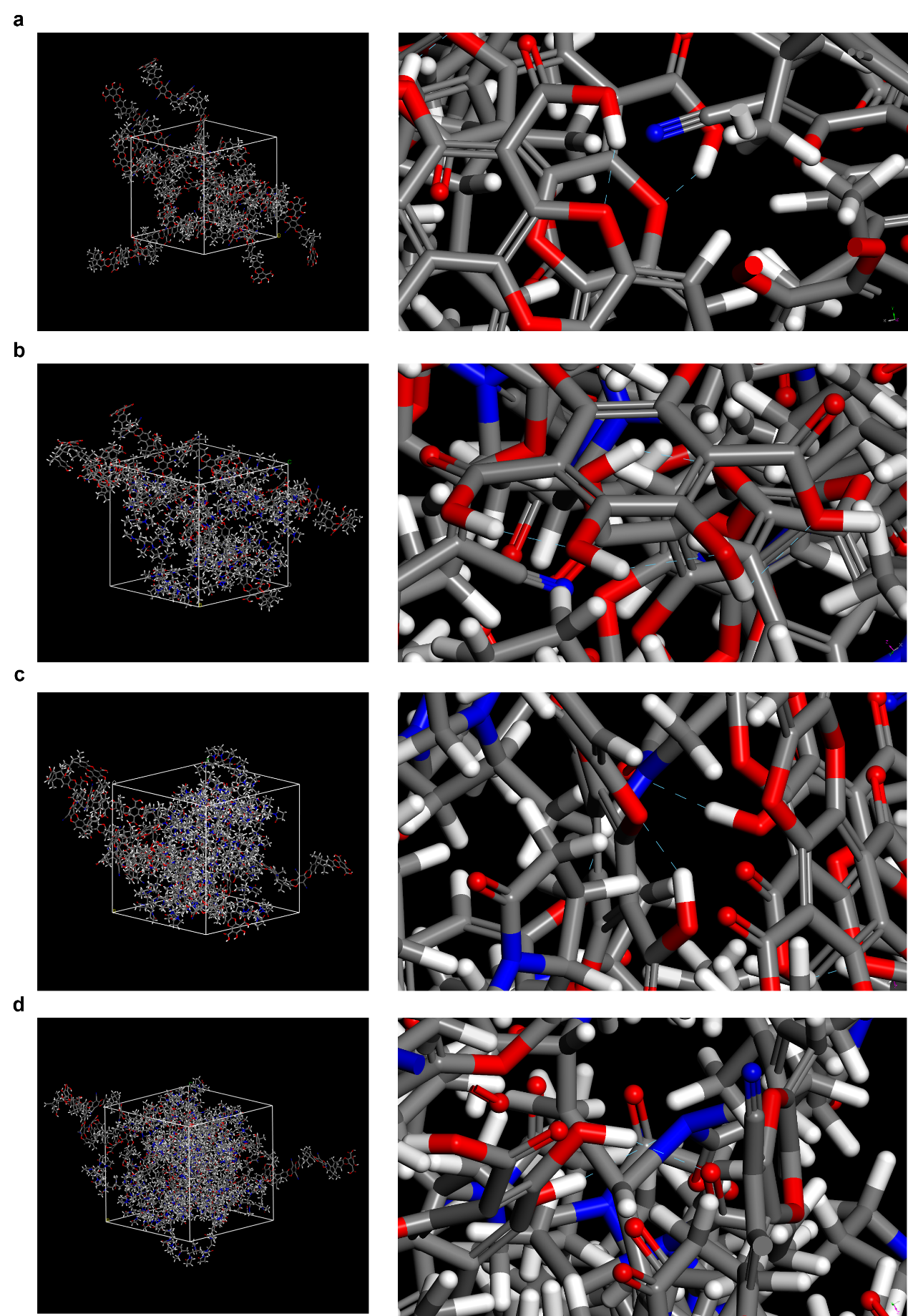


**Supplementary Figure 9.** Chain model (left) and the hydrogen bonding distribution (right) calculated by molecular dynamics simulations in the unit cells of a) cPIM-1, b) 0.5PVP/cPIM-1, c) 0.6PVP/cPIM-1 and d) 0.7PVP/cPIM-1. Red: O, gray: C. blue: N, white: H.


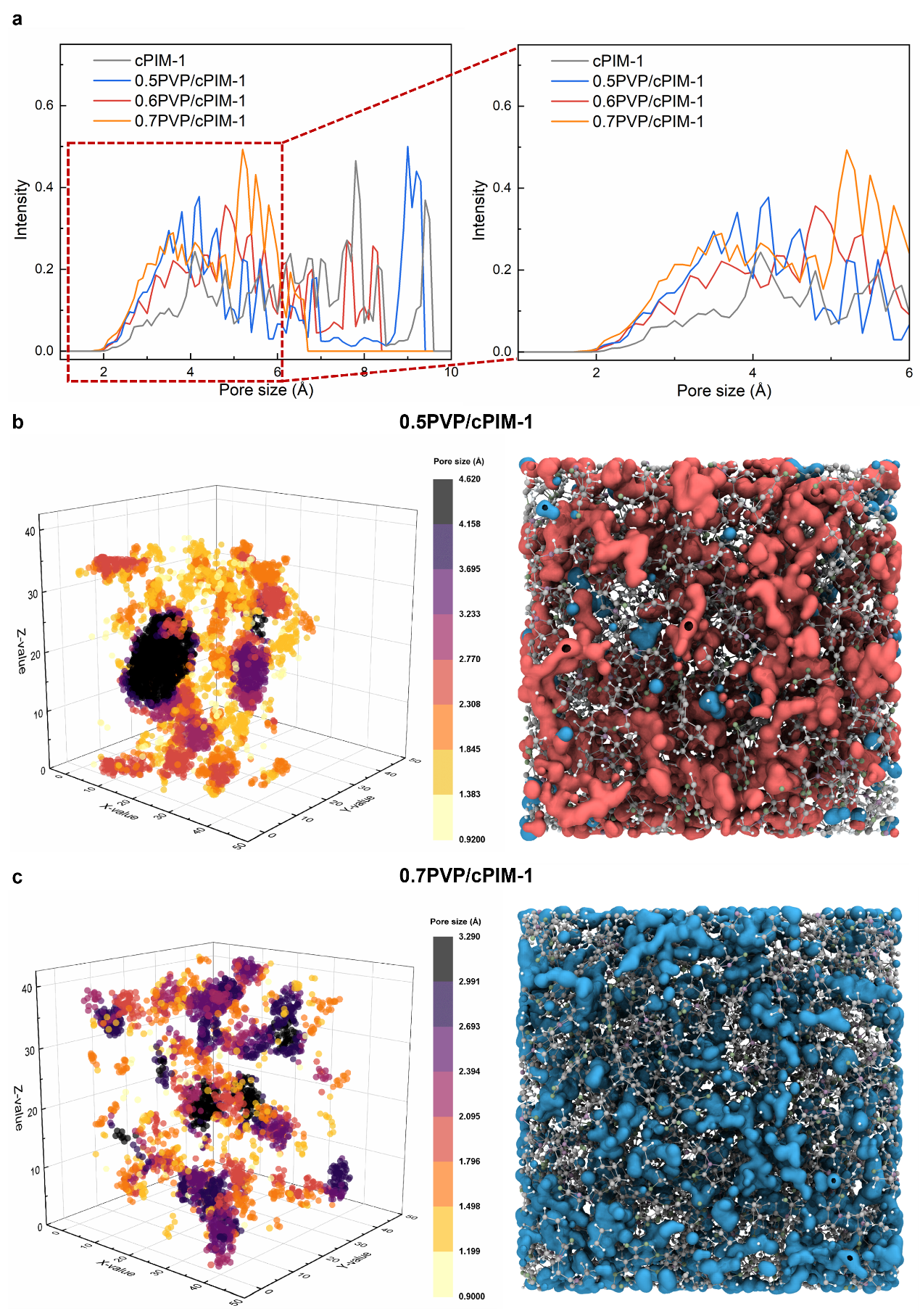


**Supplementary Figure 10.** a) Pore size distribution derived from molecular dynamic simulation based on Voronoi decomposition (0.85 Å molecular probe). 3D pore size distribution in the range of 0-5 Å (left) and the amorphous unit cell (right) of b) 0.5PVP/cPIM-1 and c) 0.7PVP/cPIM-1 membrane. Cyan indicates isolated micropores, while Cardinal red indicates interconnected micropores.

**
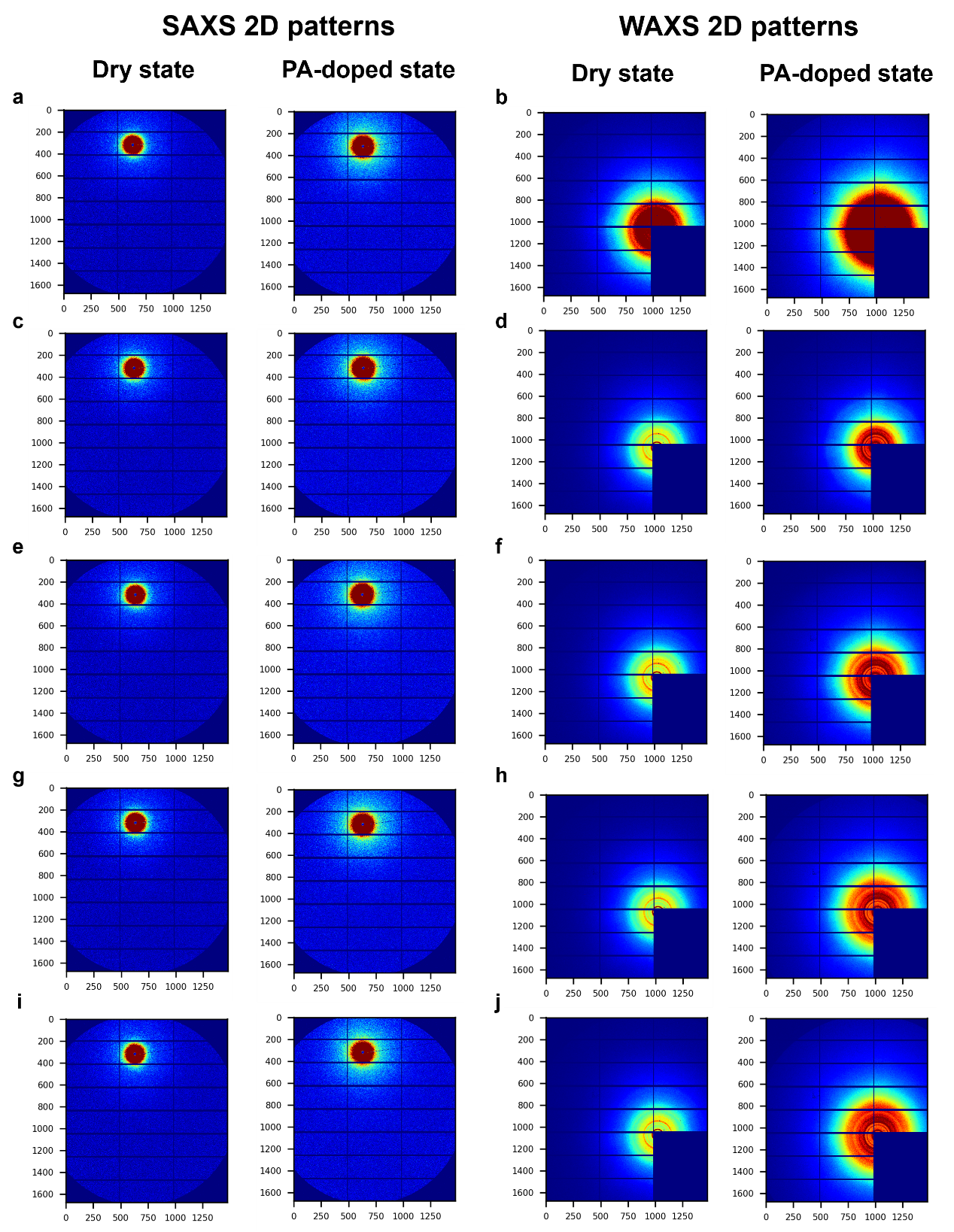
**

**Supplementary Figure 11.** SAXS and WAXS 2D patterns in dry state and PA-doped state of a,b) 0.6PVP/PES, c,d), cPIM-1 e,f) 0.5PVP/cPIM-1 and g,h) 0.6PVP/cPIM-1 and i,j) 0.7PVP/cPIM-1. X-axis and Y-axis: pixels.


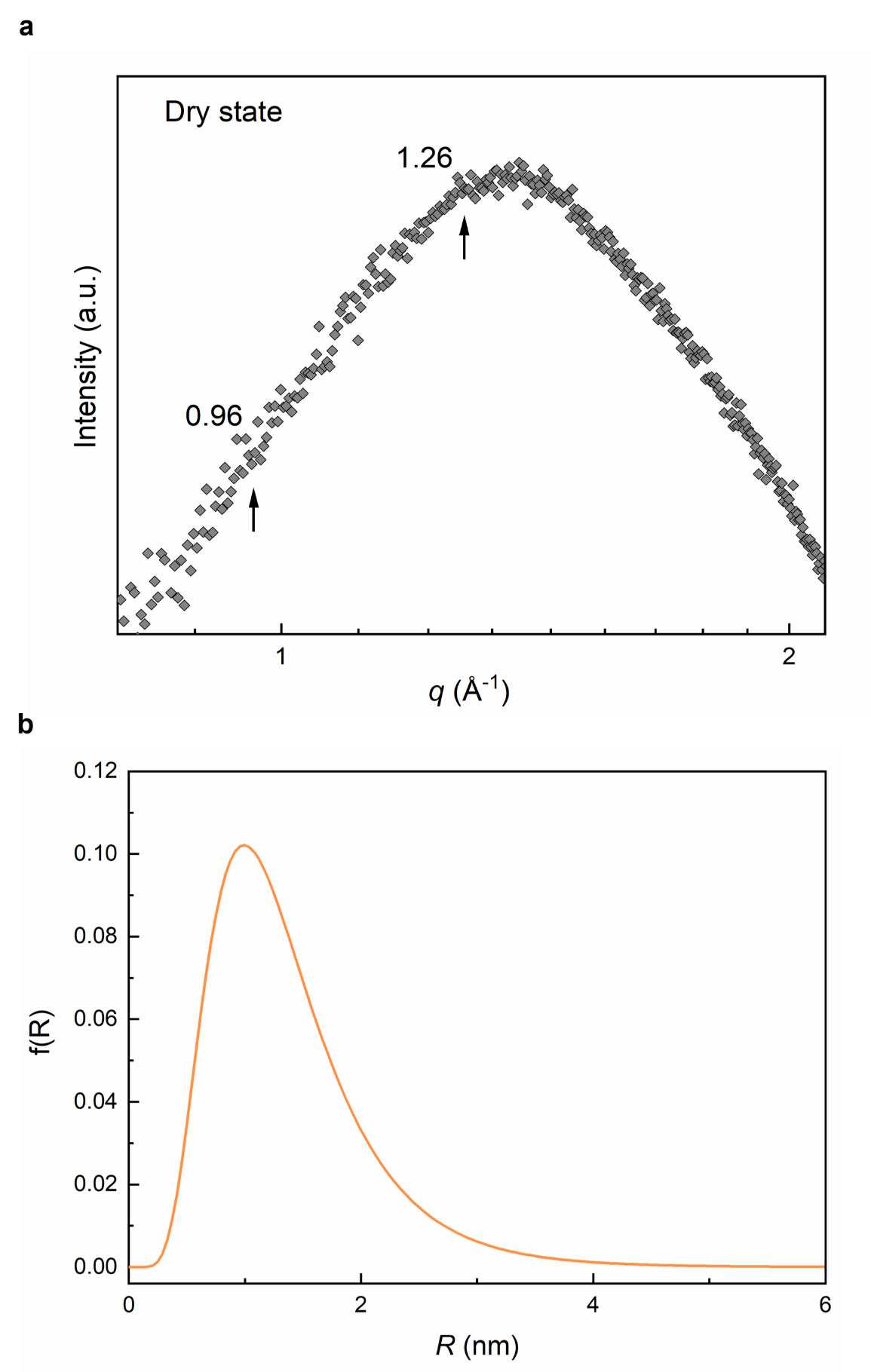


**Supplementary Figure 12.** a) Dry-state WAXS enlarged view of cPIM-1 sample, b) the scatterers size distribution of 0.7PVP/cPIM-1 sample.


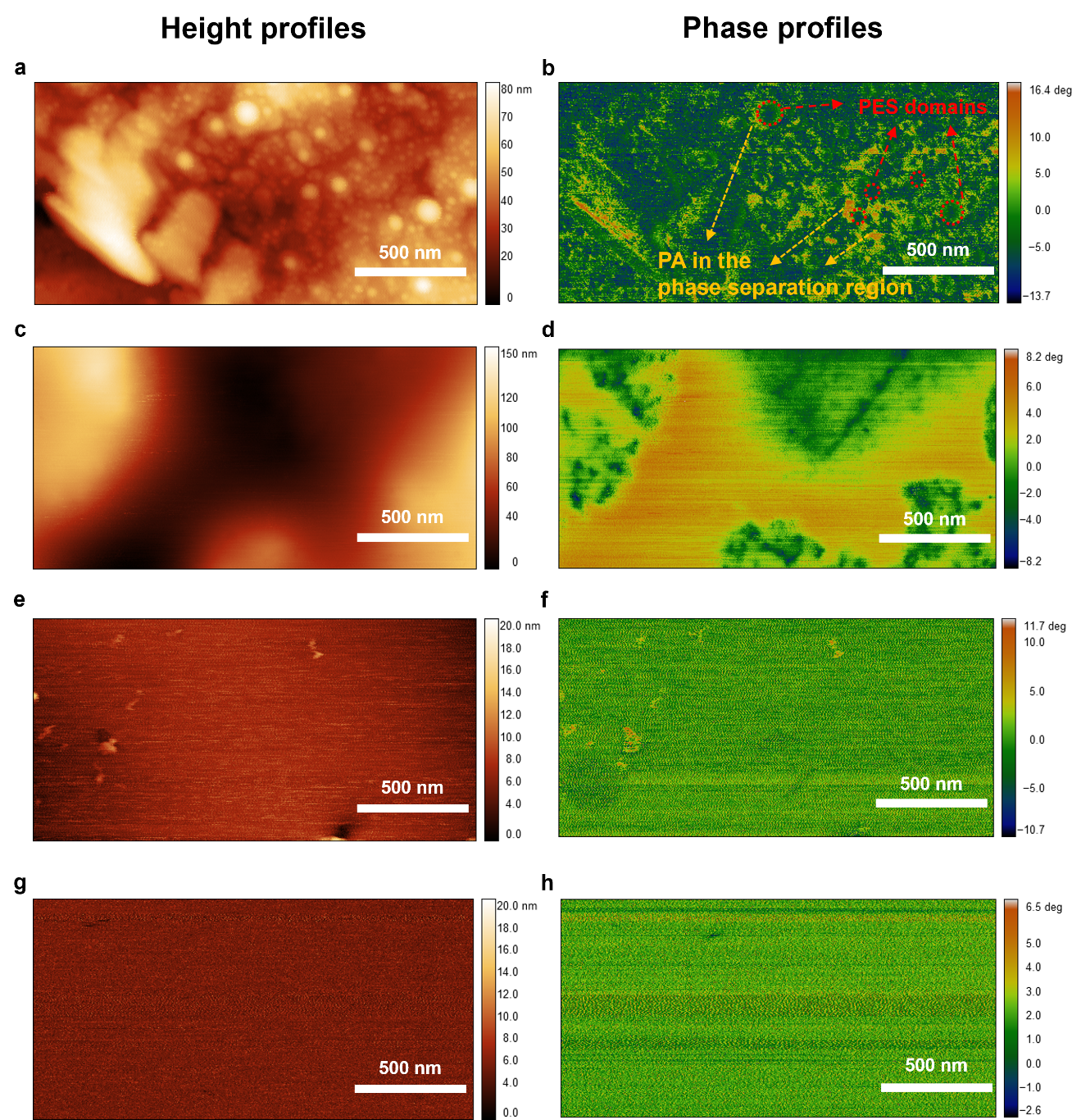


**Supplementary Figure 13.** AFM Height and phase profiles of PA-doped membranes. a,b) 0.6PVP/PES, c,d) 0.5PVP/cPIM-1, e,f) 0.6PVP/cPIM-1, g,h) 0.7PVP/cPIM-1.


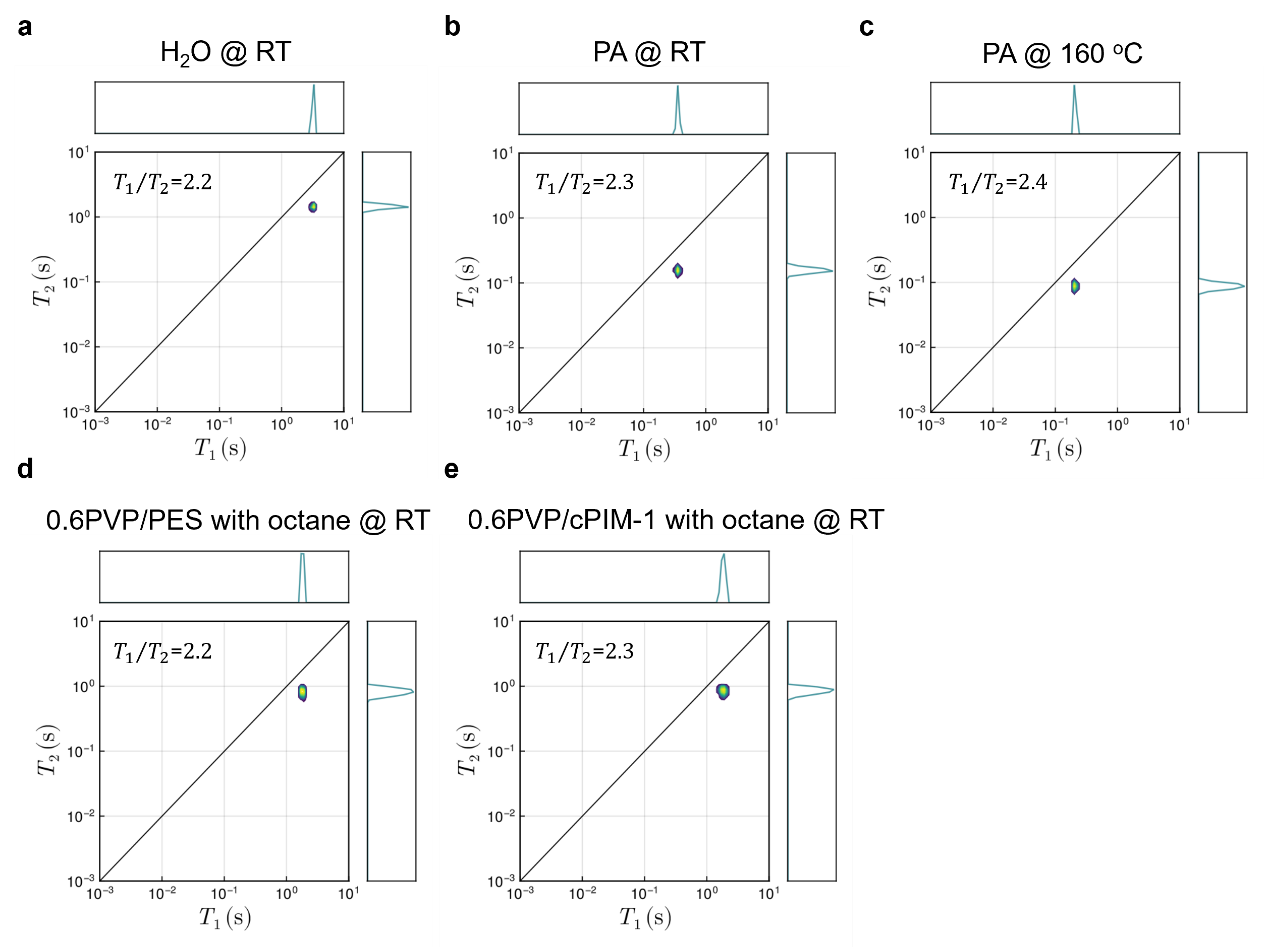


**Supplementary Figure 14.** $T_{1}-T_{2}$ relaxation correlation 2D plots of a) water at room temperature, PA b) at room temperature, c) at 160 ^o^C, d) 0.6PVP/PES and 0.6PVP/cPIM-1 for octane at room temperature.


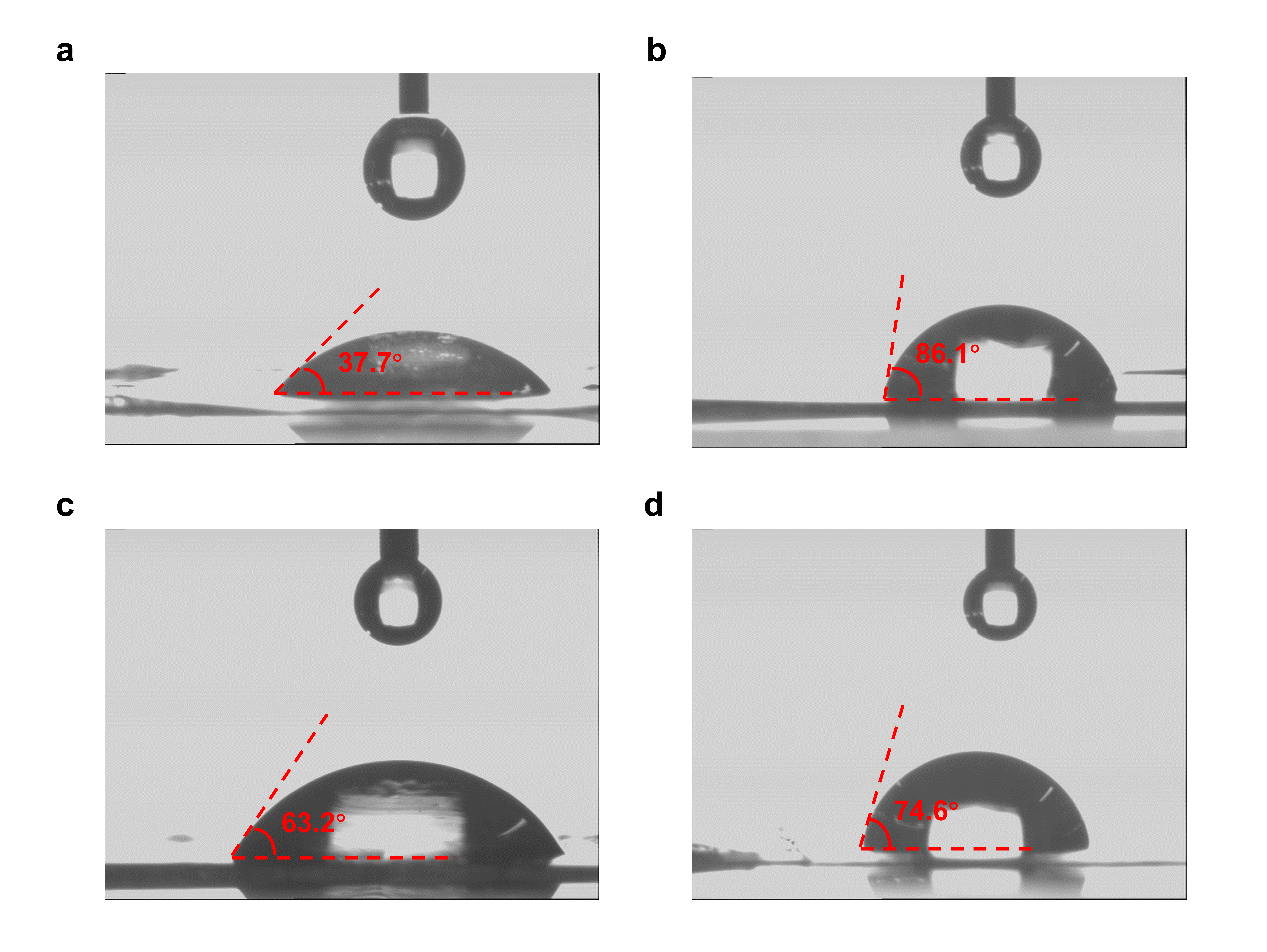


**Supplementary Figure 15.** Digital photographs of water contact angles of a) 0.6PVP/PES, b) cPIM-1, c) PBI and d) 0.6PVP/cPIM-1.





**Supplementary Figure 16.** OCV curves during fuel cell pre-heating (heating from room temperature and reaching 160 ^o^C at 1200s, then maintain 300s).


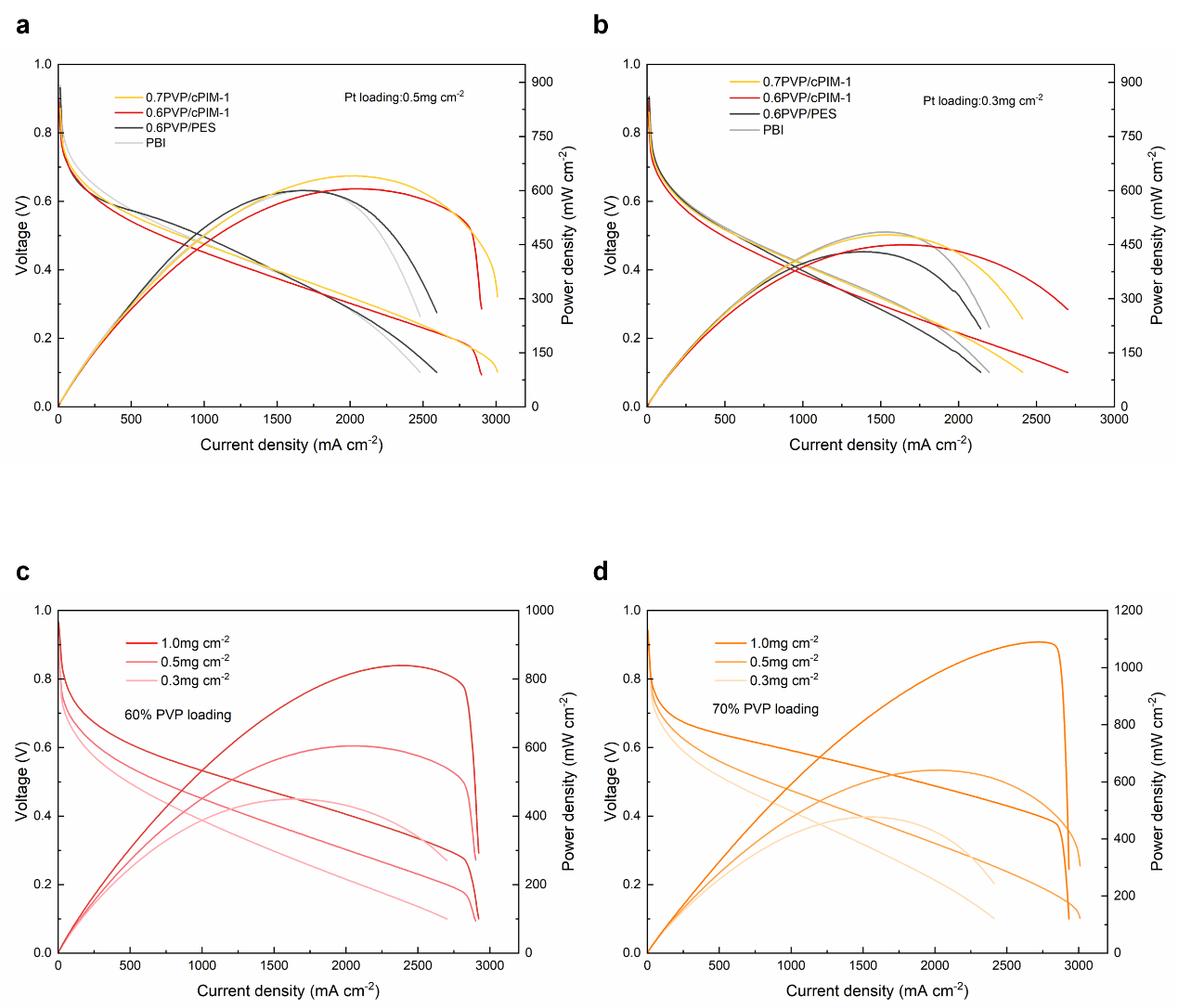


**Supplementary Figure 17.** Polarization curves and power density curves of different MEAs with a) 0.3 mg cm^-2^ Pt loading and b) 0.5 mg cm^-2^ Pt loading, different Pt loading in c) 0.6PVP/cPIM-1, d) 0.7PVP/cPIM-1. (160 ℃, anode: 100 mL min^-1^ hydrogen, 1 mg cm^-2^ Pt; Cathode: 100 mL min^-1^ oxygen, 1 mg cm^-2^ Pt).





**Supplementary Figure 18.** Constant current density durability test of 0.6PVP/PES (160 ℃, anode: 100 mL min^-1^ hydrogen, 1 mg cm^-2^ Pt; Cathode: 100 mL min^-1^ oxygen, 1 mg cm^-2^ Pt).


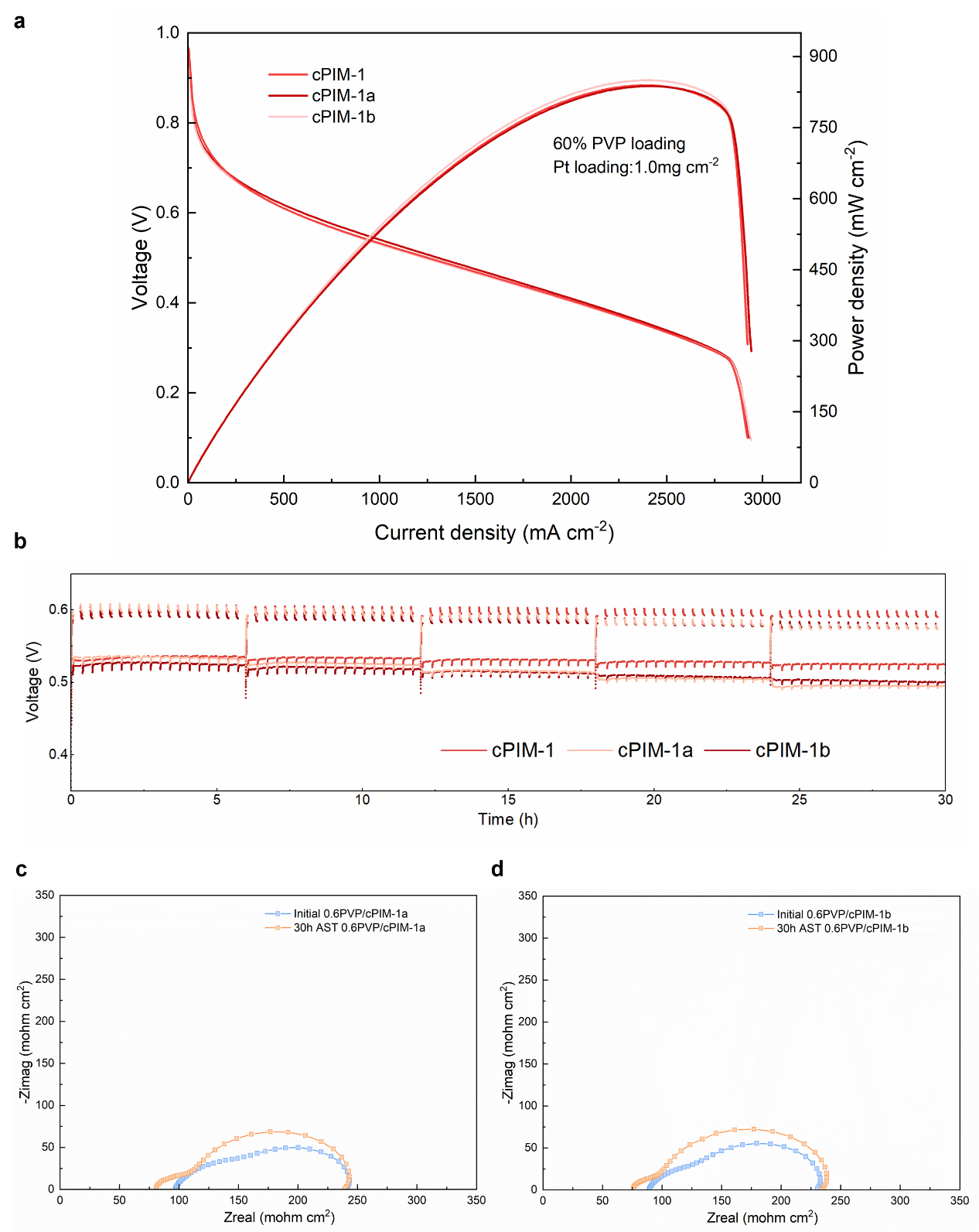


**Supplementary Figure 19.** HT-PEMFCs performance of cPIM-1 composite membranes with different hydrolysis times. a) polarization curves and power density curves, b) AST process, c,d) fitted EIS Nyquist curves before and after AST (160 ℃, anode: 100 mL min^-1^ hydrogen, 1 mg cm^-2^ Pt; Cathode: 100 mL min^-1^ oxygen, 1 mg cm^-2^ Pt).

**
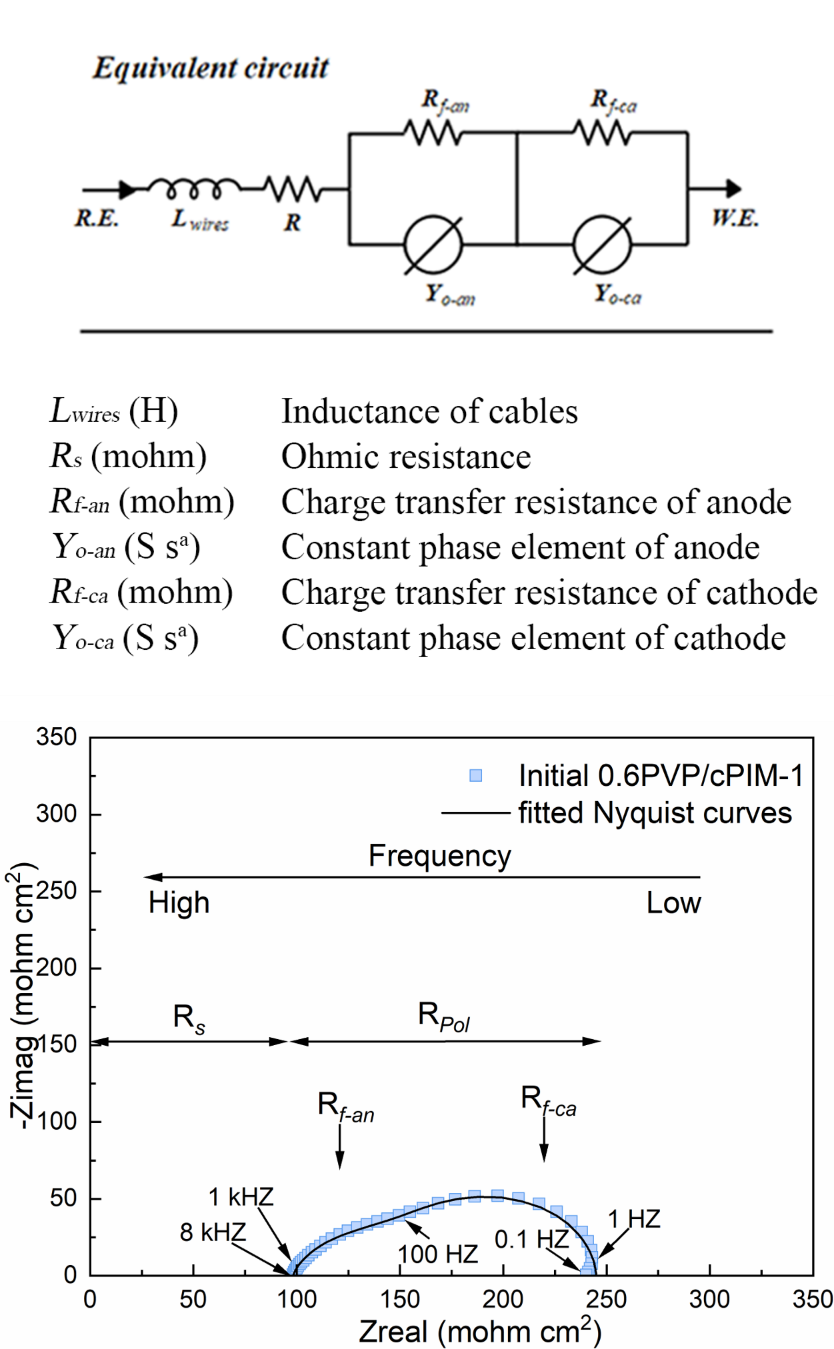
**

**Supplementary Figure 20.** The equivalent circuit, definition of parameters and EIS data fitting schematic.

**

**

**Supplementary Figure 21.** EIS fitted Nyquist curves of 0.5PVP/cPIM-1 and 0.6PVP/cPIM-1 after 210h AST (160 ℃, anode: 100 mL min^-1^ hydrogen, 1 mg cm^-2^ Pt; Cathode: 100 mL min^-1^ oxygen, 1 mg cm^-2^ Pt).


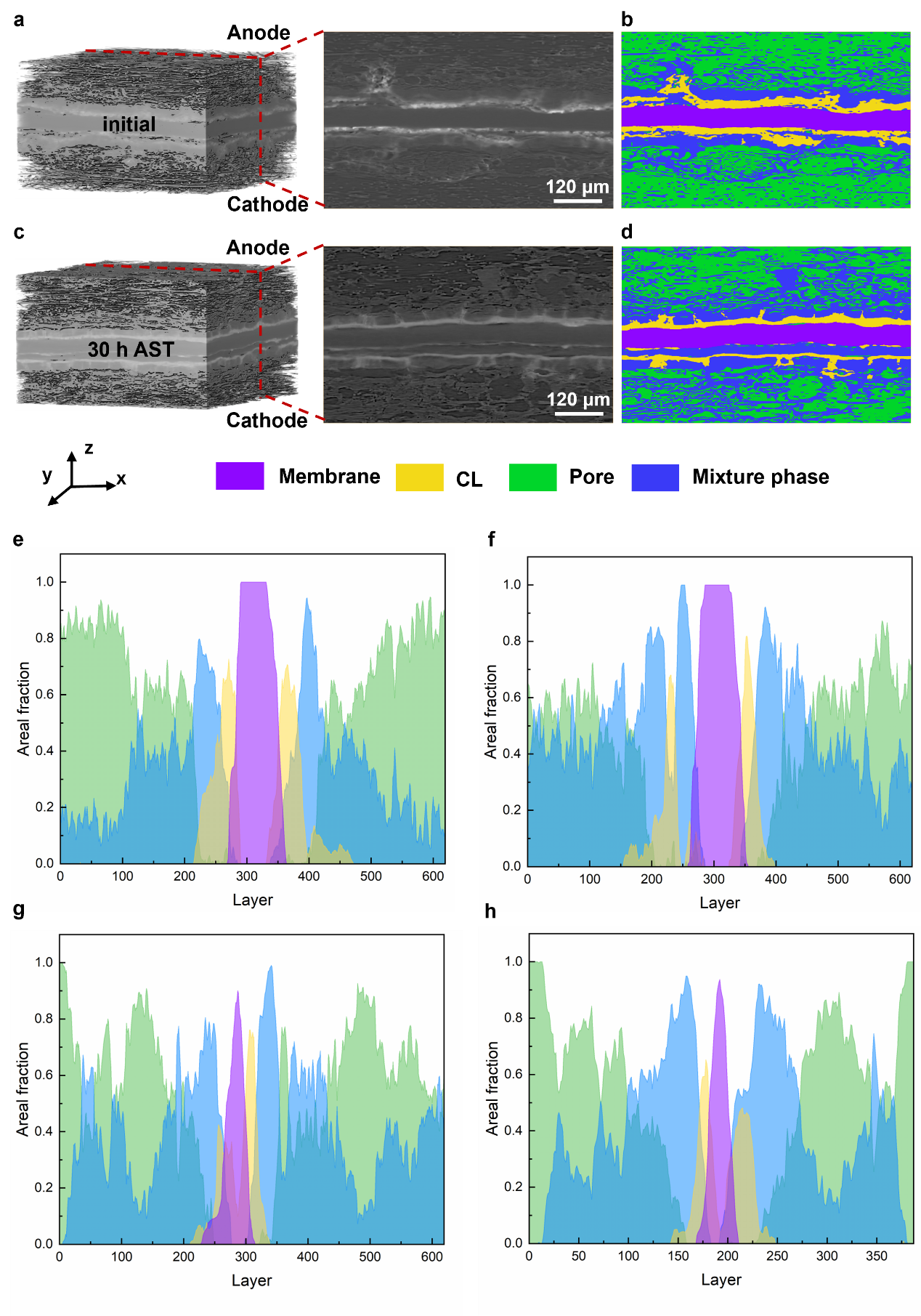


**Supplementary Figure 22.** 3D X-ray CT segmentation and the layer orthoslice of a,b) initial, c,d) 30h AST PBI. Slice-by-slice plots of area fraction in the Z-direction of e,f) initial and 30h AST PBI and g,h) initial and 30h AST 0.6PVP/PES.

**Supplementary Table 1** Details of PIM-1 synthesized from step-growth polymerizations and characterization results: Solvent, Polymer scale, Polymerization temperature, time, yield, branching, weight-average molar mass (M_w_), number-average molar mass (M_n_), dispersity (Đ) and intrinsic viscosity.

| **Sample** |  | | | | | **^1^H NMR analysis** | **Triple detector GPC analysis** | | | |
| --- | --- | --- | --- | --- | --- | --- | --- | --- | --- | --- |
|  | **Solvent mixture (2:1 by volume), 30% extra at minute 27 and 36** | **Polymer scale (TTSBI: TFTPN) (mmol)** | **Set temperature**  **(°C)** | **Time**  **(min)** | **Yield**  **(%)** | **Branching**  **(%)** | ***M*_w_**  **(kg mol^-1^)** | ***M_n_***  **(kg mol^-1^)** | ***Đ*** | **Intrinsic Viscosity**  **(cm^3^ g^-1^)** |
| PIM-1 | DMAc/  toluene | 50:50 | 160 | 45 | 85 | 7.7 | 399 | 139 | 2.9 | 55.4 |
| PIM-1a | DMAc/  toluene | 51:50 | 160 | 60 | 88 | 6.1 | 118 | 66.5 | 1.8 | 32.2 |
| PIM-1b | DMAc/  toluene | 50:50 | 160 | 45 | 92 | 6.8 | 186 | 118 | 1.6 | 46.0 |

**Supplementary Table 2** Elemental analyses of PIM-1 and cPIM-1, and conversion of -COOH calculations.

| **Polymer** | **Hydrolysis time (h)** | **C (%)** | **H (%)** | **N (%)** | **N/C ratio** | **Conversion (%)*** |
| --- | --- | --- | --- | --- | --- | --- |
| Theor. PIM-1 | - | 75.64 | 4.38 | 6.08 | 0.080 | - |
| Theor. cPIM-1 | - | 69.90 | 4.45 | 0 | - | 100 |
| PIM-1 | - | 74.40 | 4.29 | 6.19 | 0.083 | - |
| PIM-1a | - | 73.52 | 4.32 | 6.14 | 0.083 | - |
| PIM-1b | - | 74.44 | 4.35 | 5.99 | 0.080 | - |
| cPIM-1 | 24 | 67.06 | 4.71 | 2.22 | 0.033 | 60 |
| cPIM-1a | 18 | 68.75 | 4.46 | 2.32 | 0.034 | 60 |
| cPIM-1b | 36 | 63.81 | 4.44 | 1.13 | 0.018 | 78 |

* Hydrolysis conversion was calculated based on N/C ratio change through the equation:

$$Conversion=\frac{{(\frac{N}{C}ratio)}_{PIM-1}-{(\frac{N}{C}ratio)}_{cPIM-1}}{{(\frac{N}{C}ratio)}_{PIM-1}}\times100\%$$

**Supplementary Table 3** composite membranes naming and its composition.

| **Composite membranes** | **Composition** |
| --- | --- |
| 0.6PVP/PES | 60 wt.% PVP and 40 wt.% PES |
| 0.5PVP/cPIM-1 | 50 wt.% PVP and 50 wt.% cPIM-1 |
| 0.6PVP/cPIM-1 | 60 wt.% PVP and 40 wt.% cPIM-1 |
| 0.7PVP/cPIM-1 | 70 wt.% PVP and 30 wt.% cPIM-1 |
| 0.6PVP/cPIM-1a | 60 wt.% PVP and 40 wt.% cPIM-1a |
| 0.6PVP/cPIM-1b | 60 wt.% PVP and 40 wt.% cPIM-1b |

**Supplementary Table 4** The acid uptake, proton conductivity, volume and area swelling, and ADL of membrane samples.

| **Membranes** | **Thickness (μm)** | **Proton conductivity (mS cm^-1^)** | **Acid Uptake (wt. %)** | **Volume Swelling (%)** | **Area Swelling (%)** | **ADL**  **(mol)** |
| --- | --- | --- | --- | --- | --- | --- |
| PIM-1 | 59.8 | - | - | - | - | - |
| cPIM-1 | 62.3 | - | 5.3 | - | - | - |
| PBI | 60.2 | 9.9 | 308.6 | 186.1 | 56.0 | 9.7 |
| 0.6PVP/PES | 60.7 | 12.0 | 354.5 | 211.1 | 60.9 | 6.7 |
| 0.5PVP/cPIM-1 | 66.1 | 8.7 | 276.3 | 188.4 | 64.1 | 6.2 |
| 0.6PVP/cPIM-1 | 64.5 | 13.3 | 370.6 | 225.7 | 69.0 | 6.9 |
| 0.7PVP/cPIM-1 | 65.0 | 21.3 | 450.1 | 247.7 | 96.0 | 7.3 |

**Supplementary Table 5** SAXS fitting results of PA-doped state membrane samples.

| **The Guinier approximation^a^** | | | |
| --- | --- | --- | --- |
| **Sample** | $\boldsymbol{\alpha}$ | $\boldsymbol{R}_{\boldsymbol{\alpha}}$**(nm)** | $\boldsymbol{I}_{\boldsymbol{0}}$ |
| cPIM-1 | 3.8 | 5.8 | 0.04 |
| 0.5PVP/cPIM-1 | 3.6 | 1.6 | 0.02 |
| 0.6PVP/cPIM-1 | 3.1 | 2.7 | 0.08 |
| 0.6PVP/PES | 3.6 | 27.9 | 0.05 |
| **Polydisperse system of spherical particles and log-normal distribution^b^** | | | |
| **Sample** | $\boldsymbol{\alpha}$ | $\boldsymbol{R}$**(nm)** | $\boldsymbol{N}_{\boldsymbol{0}}$ |
| 0.7PVP/cPIM-1 | 2.8 | 0.3-4.2^c^ | 1.32 |

a The Guinier approximation is based on the following equation^[1]^:

$$\boldsymbol{I=}\boldsymbol{I}_{\boldsymbol{0}}\boldsymbol{exp(-}\frac{\boldsymbol{R}_{\boldsymbol{a}}^{\boldsymbol{2}}\boldsymbol{q}^{\boldsymbol{2}}}{\boldsymbol{3}}\boldsymbol{)+}\frac{\boldsymbol{A}}{\boldsymbol{q}^{\boldsymbol{\alpha}}}\boldsymbol{+B}$$

Where $I$ is the scattering intensity of the sample, $I_{0}$ is the pre-factor, which is directly proportional to the number of scatterers, $R_{\alpha}$ is the radius of gyration, $q$ is momentum transfer $\alpha$ is the porod exponent, when $\alpha$ = 4, the scatterer corresponds to a perfectly smooth, compact surface, when α ranges of 3-4, the scatterer is dense with a rough surface, and when α ranges 1.5-3, the scatterer represents mass fractal, $A$ is scaling factor and $B$ is the constant background term.

b The polydisperse system of spherical particles and log-normal distribution are based on the following equations^[1,2]^:

$\boldsymbol{I=}\boldsymbol{I}_{\boldsymbol{sphere}}\boldsymbol{+}\frac{\boldsymbol{A}}{\boldsymbol{q}^{\boldsymbol{\alpha}}}\boldsymbol{+B}$ (1)

$\boldsymbol{I}_{\boldsymbol{spher}\boldsymbol{e}}\boldsymbol{=}\int_{\boldsymbol{0}}^{\boldsymbol{\infty}} \boldsymbol{\Delta}\boldsymbol{\rho}^{\boldsymbol{2}}\boldsymbol{V}^{\boldsymbol{2}}\boldsymbol{(}\boldsymbol{R}\boldsymbol{)}\boldsymbol{f}\boldsymbol{(}\boldsymbol{R}\boldsymbol{)}\boldsymbol{F}^{\boldsymbol{2}}\boldsymbol{(}\boldsymbol{q}\boldsymbol{,}\boldsymbol{R}\boldsymbol{)}\mathbf{d}\boldsymbol{R}$ (2)

$\boldsymbol{F}\left( \boldsymbol{q,R} \right)\boldsymbol{=3}\frac{\sin\left( \boldsymbol{qR} \right)\boldsymbol{-qR}\cos\left( \boldsymbol{qR} \right)}{\left( \boldsymbol{qR} \right)^{\boldsymbol{3}}}$ (3)

$\boldsymbol{f}\left( \boldsymbol{R} \right)\boldsymbol{=}\frac{\boldsymbol{N}_{\boldsymbol{0}}}{\boldsymbol{\sigma}\sqrt{\boldsymbol{2}\boldsymbol{\pi}}}\boldsymbol{exp}\left[ \boldsymbol{-}\frac{\boldsymbol{1}}{\boldsymbol{2}\boldsymbol{\sigma}^{\boldsymbol{2}}}{\boldsymbol{(}\ln\boldsymbol{R}\boldsymbol{-}\ln\boldsymbol{R}_{\boldsymbol{0}}\boldsymbol{)}}^{\boldsymbol{2}} \right]$ (4)

Where $I_{sphere}$ is the scattering intensity of sphere, ∆ρ is the scattering length density (SLD) contrast between the scatterer and the matrix, $R$ is the radius of sphere, $V(R)$ is the volume of a sphere, $f\left( R \right)$ is the size distribution function of the spheres, $F\left( q,R \right)$ is the form factor for a sphere, $N_{0}$ is the number density (the total number of particle per unit volume), $\sigma$ is the standard deviation of the distribution in log-space.

c Obtained from the size distribution of the sample (see Supplementary Figure 12. c).

**Supplementary Table 6** Comparison and summary of the fuel cell performance based on PVP-based and PIM-based leading membranes.

| **Membrane** | **PPD**  **(mW cm^-2^)** | **Pt loading**  **(mg cm^-2^)** | **Feed gas**  **(mL min^-1^)** | **Durability test** | | **Ref.** |
| --- | --- | --- | --- | --- | --- | --- |
|  |  |  |  | **Test Condition** | **Voltage degradation (mV h^-1^)** |  |
| 0.7PVP/cPIM-1 | 1090.0 (160^o^C) | 1.0 | H_2_/O_2_,  100/100,  0 % RH | AST process: 4 min at 0.6 A cm^-2^ and 16 min 1.0 A cm^-2^, then 10min OCV, 160 ^o^C | 1.19 (0.050*) at 0.6 A cm^-2^  1.53 (0.063) at 1.0 A cm^-2^ | This work |
|  | 641.6  (160 ^o^C) | 0.5 |  | - | - |  |
|  | 477.1  (160 ^o^C) | 0.3 |  | - | - |  |
| 0.6PVP/cPIM-1 | 839.4  (160 ^o^C) | 1.0 |  | AST process: 4 min at 0.6 A cm^-2^ and 16 min 1.0 A cm^-2^, then 10min OCV,160 ^o^C | 0.058 (0.0002) at 0.6 A cm^-2^,  0.086 (0.0003) at 1.0 A cm^-2^ |  |
|  | 605.0  (160 ^o^C) | 0.5 |  | - | - |  |
|  | 449.6  (160 ^o^C) | 0.3 |  | - | - |  |
| **PVP-based** | | | | | | |
| P/0.25SN/PA | 495  (150 ^o^C) | 0.75 | H_2_/O_2_,  100/100,  0 % RH | constant voltage, 0.6 V 150 °C | 1.01-2.83 mA cm^-2^ h^-1^ | ^[3]^ |
| PBI-g-PVP 30 | 1312  (160 ^o^C) | 1.0 | H_2_/O_2_,  200/200,  0 % RH | AST process: 4 min at 0.6 A cm^-2^ and 16 min 1.0 A cm^-2^, then 5 min OCV, 160 ^o^C | 2.96 (0.59) at 1.0 A cm^-2^ | ^[4]^ |
| PES-PVP 80% | 850  (180 ^o^C) | 0.5 | H_2_/O_2_,  150/150,  0 % RH | constant voltage, 0.6 V, at 180 °C | 0.5 mA cm^-2^ h^-1^ | ^[5]^ |
| PA/PES-PVP | - | 0.8-1.0 | H_2_/Air,  100/500,  0 % RH | constant current density, 0.2 A cm^-2^, at 150 °C | 0.0097 at 0.2 A cm^-2^ | ^[6]^ |
| porous PES/PVP | 454  (180 ^o^C) | 0.5 | H_2_/O_2_,  150/150,  0 % RH | constant current density, 0.2 A cm^−2^ at 150 °C | - | ^[7]^ |
| P/CN-0.5/PA | 634  (180 ^o^C) | 0.4 | H_2_/O_2_,  150/150,  0 % RH | constant voltage, 0.6 V, at 160 °C | - | ^[8]^ |
| C-PVP-13.7%/PA | 729.4  (160 ^o^C) | 0.45 | H_2_/O_2_,  80/160,  0 % RH | constant voltage, 0.6 V, at 150 °C | - | ^[9]^ |
| PWA–NH_2_–HMS 10/PES-PVP | 420  (180) | 0.26 | H_2_/O_2_,  50/100,  0 % RH | constant current density, 0.2 A cm^−2^ at 200 °C | 6.19 | ^[10]^ |
| PES-PVP-0.5% BN | 359  (160 ^o^C) | 0.65 | H_2_/O_2_,  200/200,  0 % RH | constant current density, 0.3 A cm^−2^ at 160 °C | - | ^[11]^ |
| PES-PVP/PTFE-5 | 607  (180 ^o^C) | 0.5 | H_2_/O_2_,  150/150,  0 % RH | - | - | ^[12]^ |
| PVDF-PVP 80 | 530  (180 ^o^C) | 0.5 | H_2_/O_2_,  150/150,  0 % RH | constant voltage (0.5 V), at150 °C, 160 °C | - | ^[13]^ |
| **PIM-based** | | | | | | |
| OPBI/L-PIM 10 | 438  (160 ^o^C) | 0.6 | H_2_/O_2_,  300/150,  0 % RH | - | - | ^[14]^ |
| PIM/TB9 | 563  (160 ^o^C) | 0.5 | H_2_/O_2_,  100/100,  0 % RH | AST process: a step-V protocol from 1 V to 0.15 V, scan rate: 50 mV min^-1^，voltage is recorded at 0.3 A cm^-2^, 80^o^C | - | ^[15]^ |
| NPBI/PIM-BM-15 | 565  (160 ^o^C) | 0.5 | H_2_/O_2_,  200/200,  0 % RH | constant current density ,0.3 A cm^-2^，80 ℃ | 0.0347 | ^[16]^ |
| PTP-10cPIM/PA | 694  (160 ^o^C) | 1.0 | H_2_/O_2_,  400/400,  0 % RH | constant current density ,0.15 A cm^-2^，140 ℃ | 0.53 | ^[17]^ |
| DMBP-TB/50%PEKC/149%PA | 536  (180 ^o^C) | 0.7 | H_2_/O_2_,  60/30,  0 % RH | - | - | ^[18]^ |
| DMBP-TB/PA | 815  (160 ^o^C) | 0.5 | H_2_/O_2_,  200/200,  0 % RH | AST process: a step-V protocol from 1 V to 0.15 V, scan rate: 25 mV min^-1^, then on/off cycle 10min, 40 ^o^C | - | ^[19]^ |
| **PBI-based** | | | | | | |
| porous OPBI | 485.3  (160 ^o^C) | 0.65 | H_2_/O_2_,  200/200,  0 % RH | constant current density ,0.3 A cm^-2^，160 ℃ | 5.42 | ^[20]^ |
| DC-PBI-G | 1386  (160 ^o^C) | 1.0 | H_2_/O_2_,  -  0 % RH | constant current density of 0.2 A cm^−2^,  220^o^C | 0.27 | ^[21]^ |
| p-OPBI-ATMP | 980  (160 ^o^C) | 1.2 ± 0.1 | H_2_/O_2_,  150/200,  0 % RH | constant current density of 0.2 A cm^−2^,  160^o^C | 0.00546 | ^[22]^ |
| three-layer-OPBI | 604.6  (160 ^o^C) | 1.0 | H_2_/O_2_,  80/160,  0 % RH | constant current density of 0.2 A cm^−2^,  160^o^C | 0.73 | ^[23]^ |
| PBI-SLG | 399  (150 ^o^C) | 1.0 | H_2_/O_2_,  100/100,  0 % RH | AST process: 4 min at 0.6 A cm^-2^ and 16 min 1.0 A cm^-2^, then 10min OCV, 150 ^o^C | 1.71 at 1.0 A cm^-2^  1.68 at 0.6 A cm^-2^ | ^[24]^ |
| 40 %-OPBI | 1090.5  (160 ^o^C) | 0.6 | H_2_/O_2_,  80/160,  0 % RH | constant current density of 0.2 A cm^−2^,  160 ^o^C | 0.0132 | ^[25]^ |
| CsH_5_(PO_4_)_2_ -doped PBI | 410  (160 ^o^C) | 1.0 | H_2_/O_2_,  200/200,  0 % RH | constant current density of 0.2 A cm^−2^,  160 ^o^C | 0.17 | ^[26]^ |

**Supplementary Table 7** $R_{Pol}$of different MEAs before and after AST obtained from equivalent circuits.

| **Samples** | $\boldsymbol{R}_{\boldsymbol{P}\boldsymbol{ol}}$ **(mΩ cm^2^)** | | |
| --- | --- | --- | --- |
|  | **Initial** | **30h** | **210h** |
| PBI | 166.1 | 203.2 | - |
| 0.6PVP/PES | 183.7 | 277.6 | - |
| 0.5PVP/cPIM-1 | 196.7 | 186.1 | 160.1 |
| 0.6PVP/cPIM-1 | 148.5 | 149.9 | 158.9 |
| 0.7PVP/cPIM-1 | 137.0 | 155.0 | - |

**Supplementary Table 8** $R_{s}, R_{f-an} and R_{f-ca}$ of different MEAs before and after AST obtained from equivalent circuits.

| **Samples** | $\boldsymbol{R}_{\boldsymbol{s}}$ **(mΩ cm^2^)** | | | $\boldsymbol{R}_{\boldsymbol{f}\boldsymbol{-}\boldsymbol{an}}$ **(mΩ cm^2^)** | | | $\boldsymbol{R}_{\boldsymbol{f}\boldsymbol{-}\boldsymbol{ca}}$ **(mΩ cm^2^)** | | |
| --- | --- | --- | --- | --- | --- | --- | --- | --- | --- |
|  | **Initial** | **30h** | **210h** | **Initial** | **30h** | **210h** | **Initial** | **30h** | **210h** |
| PBI | 111.5 | 102.7 | - | 29.7 | 59.8 | - | 136.7 | 138.1 | - |
| 0.6PVP/PES | 82.1 | 77.6 | - | 69.7 | 121.0 | - | 115.6 | 151.9 | - |
| 0.5PVP/cPIM-1 | 133.0 | 97.7 | 68.0 | 13.5 | 38.8 | 53.7 | 165.9 | 151.2 | 108.7 |
| 0.6PVP/cPIM-1 | 96.3 | 82.7 | 59.7 | 37.8 | 29.1 | 51.5 | 117.3 | 119.3 | 109.5 |
| 0.7PVP/cPIM-1 | 51.5 | 42.4 | - | 28.4 | 30.0 | - | 115.1 | 129.6 | - |

**Supplementary Table 9** GDL/MPL/PA phase fraction of different MEA samples.

| **Sample** | **Electrode** | **GDL/MPL/PA phase fraction** |
| --- | --- | --- |
| Initial 0.6PVP/cPIM-1 | Anode | 41.0% |
|  | Cathode | 38.1% |
| 30h AST 0.6PVP/cPIM-1 | Anode | 41.8% |
|  | Cathode | 41.2% |
| 210h AST 0.6PVP/cPIM-1 | Anode | 34.3% |
|  | Cathode | 34.6% |
| Initial 0.6PVP/PES | Anode | 43.1% |
|  | Cathode | 40.4% |
| 30h AST 0.6PVP/PES | Anode | 45.0% |
|  | Cathode | 42.8% |
| Initial PBI | Anode | 34.5% |
|  | Cathode | 33.5% |
| 30h AST PBI | Anode | 45.1% |
|  | Cathode | 56.3% |

**Supplementary Table 10** Proton conductivity of different membrane samples at different stages.

| **Sample** | **Proton conductivity (mS cm^-1^)** | | |
| --- | --- | --- | --- |
|  | **Initial** | **30h AST** | **210h AST** |
| PBI | 9.9 | 9.7 | - |
| 0.6PVP/PES | 12.0 | 7.7 | - |
| 0.6PVP/cPIM-1 | 13.3 | 12.1 | 10.4 |

**References**

[1] L. Peng, B. Chen, Y. Zhao, *Constr. Build. Mater.* **2020**, *262*, 120863.

[2] Q. Tian, G. Yan, G. Sun, C. Huang, L. Xie, B. Chen, M. Huang, H. Li, X. Liu, J. Wang, *Cent. Eur. J. Energ. Mater.* **2013**, *Vol. 10*.

[3] Z. Guo, J. Chen, J. J. Byun, R. Cai, M. Perez-Page, M. Sahoo, Z. Ji, S. J. Haigh, S. M. Holmes, *J. Energy Chem.* **2022**, *64*, 323.

[4] Z. Zhou, Z. Zhao, X. Yang, H. Zhai, L. Ai, J. Chen, S. Holmes, *J. Memb. Sci.* **2024**, *710*, 123135.

[5] X. Xu, H. Wang, S. Lu, Z. Guo, S. Rao, R. Xiu, Y. Xiang, *J. Power Sources* **2015**, *286*, 458.

[6] J. Zhang, Z. B. Guo, J. J. Zhang, H. N. Wang, Y. Xiang, S. P. Jiang, and S. F. Lu, *CIESC Journal* **2021**, *72*, *no. 1*, 589.

[7] Z. Guo, R. Xiu, S. Lu, X. Xu, S. Yang, Y. Xiang, *J. Mater. Chem. A* **2015**, *3*, 8847.

[8] H. Bai, H. Wang, J. Zhang, C. Wu, J. Zhang, Y. Xiang, S. Lu, *J. Memb. Sci.* **2018**, *558*, 26.

[9] H. Bai, J. Zhang, H. Wang, Y. Xiang, S. Lu, *J. Memb. Sci.* **2022**, *645*, 120194.

[10] J. Zhang, J. Liu, S. Lu, H. Zhu, D. Aili, R. De Marco, Y. Xiang, M. Forsyth, Q. Li, S. P. Jiang, *ACS Appl. Mater. Interfaces* **2017**, *9*, 31922.

[11] B. Lv, H. Yin, Z. Huang, K. Geng, X. Qin, W. Song, Z. Shao, *J. Memb. Sci.* **2022**, *653*, 120512.

[12] S. Lu, R. Xiu, X. Xu, D. Liang, H. Wang, Y. Xiang, *J. Memb. Sci.* **2014**, *464*, 1.

[13] Z. Guo, X. Xu, Y. Xiang, S. Lu, S. P. Jiang, *J. Mater. Chem. A* **2014**, *3*, 148.

[14] P. Wang, Z. Liu, X. Li, J. Peng, W. Hu, B. Liu, *Chem. Commun.* **2019**, *55*, 6491.

[15] H. Tang, G. Chao, J. Gao, Y. Shang, N. Li, K. Geng, *J. Power Sources* **2023**, *565*, 232868.

[16] T. Guo, Y. Wang, Q. Ju, S. Kang, G. Chao, X. Chen, R. Li, Z. Lv, Y. Shen, N. Li, K. Geng, *J. Memb. Sci.* **2023**, *675*, 121528.

[17] B. Liu, Y. Duan, T. Li, Y. Pang, Q. Liu, Q. Li, X. Hu, C. Zhao, *J. Memb. Sci.* **2024**, *692*, 122273.

[18] T. Wang, Y. Jin, T. Mu, T. Wang, J. Yang, *J. Memb. Sci.* **2022**, *654*, 120539.

[19] H. Tang, K. Geng, L. Wu, J. Liu, Z. Chen, W. You, F. Yan, M. D. Guiver, N. Li, *Nat. Energy 2022 72* **2022**, *7*, 153.

[20] K. Geng, H. Tang, Q. Ju, H. Qian, N. Li, *J. Memb. Sci.* **2021**, *620*, 118981.

[21] L. Zhang, M. Liu, D. Zhu, M. Tang, T. Zhu, C. Gao, F. Huang, L. Xue, *Nat. Commun. 2024 151* **2024**, *15*, 1.

[22] W. Li, W. Liu, J. Zhang, H. Wang, S. Lu, Y. Xiang, W. Li, W. Liu, J. Zhang, H. Wang, S. Lu, Y. Xiang, *Adv. Funct. Mater.* **2023**, *33*, 2210036.

[23] P. Wang, J. Peng, B. Yin, X. Fu, L. Wang, J. L. Luo, X. Peng, *J. Mater. Chem. A* **2021**, *9*, 26345.

[24] J. Chen, J. J. Bailey, L. Britnell, M. Perez-Page, M. Sahoo, Z. Zhang, A. Strudwick, J. Hack, Z. Guo, Z. Ji, P. Martin, D. J. L. Brett, P. R. Shearing, S. M. Holmes, *Nano Energy* **2022**, *93*, 106829.

[25] J. Peng, X. Fu, J. Luo, L. Wang, X. Peng, *Chem. Eng. J.* **2023**, *453*, 139609.

[26] Z. Fu, Y. Li, J. Papavasiliou, Y. Xing, L. Liu, Z. Li, L. Chen, H. Li, *Int. J. Energy Res.* **2022**, *46*, 24148.
